# The relationship between resting‐state amplitude fluctuations and memory‐related deactivations of the default mode network in young and older adults

**DOI:** 10.1002/hbm.26299

**Published:** 2023-04-13

**Authors:** Jasmin M. Kizilirmak, Joram Soch, Hartmut Schütze, Emrah Düzel, Hannah Feldhoff, Larissa Fischer, Lea Knopf, Anne Maass, Matthias Raschick, Annika Schult, Renat Yakupov, Anni Richter, Björn H. Schott

**Affiliations:** ^1^ Cognitive Geriatric Psychiatry German Center for Neurodegenerative Diseases Göttingen Germany; ^2^ Neurodidactics and NeuroLab Institute for Psychology, University of Hildesheim Hildesheim Germany; ^3^ German Centre for Higher Education Research and Science Studies Hannover Germany; ^4^ Bernstein Center for Computational Neuroscience Berlin Germany; ^5^ Medical Faculty, Institute for Cognitive Neurology and Dementia Research Otto‐von‐Guericke‐University Magdeburg Germany; ^6^ Center for Behavioral Brain Sciences Magdeburg Germany; ^7^ German Center for Neurodegenerative Diseases Magdeburg Germany; ^8^ Leibniz Institute for Neurobiology Magdeburg Germany; ^9^ Center for Intervention and Research on Adaptive and Maladaptive Brain Circuits Underlying Mental Health (C‐I‐R‐C) Jena‐Magdeburg‐Halle Germany; ^10^ Department of Psychiatry and Psychotherapy University Medical Center Göttingen Göttingen Germany

**Keywords:** aging, DMN, fMRI, long‐term memory, mPFC, precuneus, resting state, subsequent memory

## Abstract

The default mode network (DMN) typically exhibits deactivations during demanding tasks compared to periods of relative rest. In functional magnetic resonance imaging (fMRI) studies of episodic memory encoding, increased activity in DMN regions even predicts later forgetting in young healthy adults. This association is attenuated in older adults and, in some instances, increased DMN activity even predicts remembering rather than forgetting. It is yet unclear whether this phenomenon is due to a compensatory mechanism, such as self‐referential or schema‐dependent encoding, or whether it reflects overall reduced DMN activity modulation in older age. We approached this question by systematically comparing DMN activity during successful encoding and tonic, task‐independent, DMN activity at rest in a sample of 106 young (18–35 years) and 111 older (60–80 years) healthy participants. Using voxel‐wise multimodal analyses, we assessed the age‐dependent relationship between DMN resting‐state amplitude (mean percent amplitude of fluctuation, mPerAF) and DMN fMRI signals related to successful memory encoding, as well as their modulation by age‐related hippocampal volume loss, while controlling for regional grey matter volume. Older adults showed lower resting‐state DMN amplitudes and lower task‐related deactivations. However, a negative relationship between resting‐state mPerAF and subsequent memory effect within the precuneus was observed only in young, but not older adults. Hippocampal volumes showed no relationship with the DMN subsequent memory effect or mPerAF. Lastly, older adults with higher mPerAF in the DMN at rest tend to show higher memory performance, pointing towards the importance of a maintained ability to modulate DMN activity in old age.

## 1 INTRODUCTION

The default mode network (DMN; Buckner et al., 2008; Raichle et al., 2001) is typically deactivated during cognitively demanding tasks (e.g., attention or working memory tasks), and the brain regions forming the DMN show increased connectivity at rest. The DMN encompasses the medial prefrontal cortex (mPFC), the posterior cingulate cortex (PCC), and the precuneus, as well as the bilateral temporoparietal junction (TPJ). These regions have been collectively shown to be more active when focusing on internally represented information, such as during self‐reference, mind‐wandering, or long‐term and autobiographical memory retrieval (Andrews‐Hanna et al., 2014; Schilbach et al., 2012; Spreng, 2012). Particularly posterior DMN structures are activated during context‐rich episodic recall (Buckner et al., 2008; Greicius et al., 2004; Henson et al., 1999; Hu et al., 2013; Sajonz et al., 2010; Schott et al., 2005; Sreekumar et al., 2018).

Compared to young individuals, older adults typically show reduced deactivations of DMN structures like the mPFC and the precuneus during various cognitive tasks, including successful episodic encoding, working memory, or semantic categorization (Hayes et al., 2017; Persson et al., 2014; Sambataro et al., 2010). This finding is commonly thought to reflect the decreased ability of older adults to direct resources towards the task—away from spontaneous inwardly directed thought.

During long‐term memory *encoding*, reduced deactivation of DMN structures in young healthy adults is associated with later forgetting (see Kim, 2011; Maillet & Rajah, 2014, for a meta‐analysis). Remarkably, particularly in older adults with lower memory performance, several studies have reported a flip in the sense that reduced DMN deactivation (or even above‐baseline activation) predicts later *remembering* rather than forgetting (Düzel et al., 2011; Maillet & Rajah, 2014; Wang et al., 2010). This seemingly paradoxical phenomenon of reduced DMN deactivation predicting later remembering in older adults with poor behavioral memory performance constitutes a well‐replicated finding. However, it is yet unclear whether this observation is best explained by a general, task‐independent hyperactivity of the DMN in old age (Sambataro et al., 2010; Wang et al., 2010) or whether it reflects different encoding strategies that older adults adopt to compensate for age‐related deficits in hippocampus‐dependent encoding (Cabeza et al., 2005).

In the present study, we aimed to elucidate the relationship between DMN activity at rest and during successful memory encoding in young and older adults. Specifically, we aimed to assess whether there was a direct relationship between activity variation in DMN structures at rest and during successful encoding and whether such a relationship was related to age.

Although task‐unrelated DMN hyperactivity in older adults cannot be excluded as an explanation for the reduced deactivation during cognitive tasks, it seems insufficient to explain the phenomenon that—at least in a subpopulation of older adults—DMN activity predicts subsequent *remembering*. This seems to be more readily explained by a targeted compensatory encoding mechanism, which would be in line with a study by Turner and Spreng (2015) who reported functional connectivity increases between lateral PFC and DMN regions during cognitively demanding tasks in older, but not in younger adults. They suggested that this might indicate a shift in strategy by relying more on prior knowledge, supporting the default‐executive coupling hypothesis which poses that prior knowledge increasingly supports executive processes to compensate for age‐related decline (Craik & Bialystok, 2006). Indeed, especially the DMN's midline regions, especially the mPFC and precuneus, have been implicated in cognition focused on internal representations, such as self‐reference (Craik et al., 1999; Macrae et al., 2004), social processing (Meyer et al., 2019), reward‐related processing (Adcock et al., 2006; Wittmann et al., 2005), and emotion processing (Murty et al., 2010; Sambataro et al., 2010).

Notably, both lesion studies in rodents (Tse et al., 2007, 2011) and functional magnetic resonance imaging (fMRI) studies in healthy young adults (Kizilirmak et al., 2019; van Kesteren et al., 2012, 2013) have shown that the ventral mPFC plays an important role in a type of long‐term memory formation that either strongly accelerates or even shortcuts *hippocampal* memory encoding. This type of processing relies heavily on existing memory representations, that is, generalized, more abstract knowledge, and older adults tend to rely more heavily on it (Kizilirmak et al., 2021; Koutstaal & Schacter, 1997; Schacter & Norman, 1997; Webb & Dennis, 2020).

It is thus conceivable that reduced deactivations (or even activations) of midline structures during successful encoding in older adults may indicate an increasing reliance on *less hippocampus‐dependent forms of encoding*, potentially triggered by the increasing inefficiency of hippocampus‐dependent memory encoding due to age‐related hippocampal volume loss. Although this mechanism may be thought of along the lines of cognitive reserve (Cabeza et al., 2018; Stern, 2009, 2012), that is, a compensatory neurocognitive strategy, it should be noted that the strategy is not necessarily a beneficial one, as actual DMN *activations* during successful encoding have primarily been found in older adults with overall poor episodic memory performance (Maillet & Rajah, 2014).

Results from resting‐state fMRI (rs‐fMRI) studies in older adults are not entirely conclusive with respect to DMN hyperactivity. For example, in a study using independent component analysis of rs‐fMRI data, older adults were found to exhibit decreased rather than increased DMN co‐activation magnitude in the PCC, a hub of the posterior DMN (Koch et al., 2010). Furthermore, anti‐correlations of PCC activity and task‐positive networks are reduced in old age (Esposito et al., 2018). Most rs‐fMRI studies of DMN function in old age have employed functional connectivity analyses and typically report reduced connectivity between DMN regions (Badhwar et al., 2017; Binnewijzend et al., 2012; Sheline & Raichle, 2013), which accompanies the reduced task‐related deactivations and is pronounced in dementia risk states (Hafkemeijer et al., 2012). As these findings largely rely on functional connectivity, they do not exclude the possibility of undirected hyperactivity of DMN structures in older adults. To assess age‐related variation in resting‐state activity irrespective of intra‐DMN connectivity, amplitude‐based measures like the (fractional) amplitude of low‐frequency fluctuation (ALFF/fALFF; La et al., 2016) or the more recently described percent amplitude fluctuations (PerAF; Jia et al., 2020) might constitute a more appropriate measure. PerAF is a scale‐independent, sign‐free, voxel‐wise measure of the percentage of low‐frequency BOLD fluctuations relative to the mean BOLD signal intensity (Jia et al., 2020). It is therefore more comparable with classical task‐fMRI effects, which typically represent the voxel‐wise relative difference of the BOLD signal amplitudes between different experimental conditions.

Here, we investigated the relationship between age‐related hippocampal volume, DMN (de‐)activation during successful episodic encoding in a subsequent‐memory task, and DMN activity fluctuation at rest in healthy young compared to older participants. The subsequent memory paradigm employed was specifically developed to assess the age‐related functional activity deviation during memory encoding (Düzel et al., 2011). During fMRI scanning, participants encoded photographs incidentally while performing an indoor/outdoor categorization. Memory was tested 70 min later via 5‐point recognition‐confidence ratings. This rating was used for the computation of fMRI effects related to successful encoding. DMN BOLD fluctuation at rest was assessed using PerAF (Jia et al., 2019). Because PerAF allows voxel‐wise assessment of the resting‐state fMRI signal, it can be used for voxel‐wise comparison with DMN activity as assessed by a difference due to later memory contrast from task‐fMRI. As we hypothesized that a DMN involvement in episodic encoding may be due to a shift in encoding strategy due to reduced ability to rely on hippocampal encoding, we also assessed hippocampal volumes for each participant to put it in relation to reduced DMN deactivation (or hyperactivation, considering our hypothesis). Moreover, although structural differences between young and older adults are quite substantial, regional differences in grey matter volume (GMV) are rarely taken into account as a potentially confounding factor when comparing fMRI data between age groups. We, therefore, controlled for this potential source of age differences by including GMV in our models.

### 1.1 Hypotheses

**FIGURE 1:**
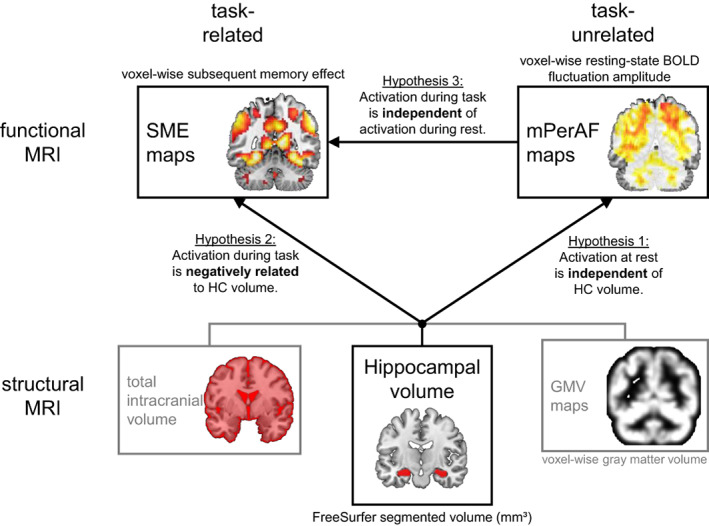
Study outline. Three main hypotheses (which mainly focus on the older participants) and involved imaging variables are presented. As grey matter volume (GMV) maps and total intracranial volume only served as covariates of no interest, they are grayed out.

Before data analysis, the hypotheses and analysis protocol were registered with the Open Science Foundation (OSF; for the original protocol see here, https://osf.io/gfw85/). An overview of the hypotheses and involved imaging variables is presented in Figure 1. The following hypotheses were formulated:

1. BOLD signal variation within the DMN *at rest*, as assessed by mPerAF, especially midline regions like mPFC and precuneus, would be independent of hippocampal volumes.
2. DMN hyperactivation *during successful encoding* was expected to be negatively related to hippocampal volumes in older adults. As long as atrophy was largely confined to MTL regions, DMN regions, especially the mPFC and precuneus, would be compensatorily recruited during encoding.
3. *Control*: DMN hyperactivation during successful encoding was hypothesized to be independent of the degree of the amplitude of BOLD signal fluctuations in the DMN at rest.

Additionally, as elaborated above, some findings suggest that, although many older adults show this pattern of reduced DMN deactivation during successful encoding, it may not actually be very beneficial for later memory performance (Maillet & Rajah, 2014). Thus, we assessed the relationship between DMN hyperactivation and later memory performance across subjects, expecting that increased DMN activity would relate to poorer memory performance.

All hypotheses were tested in data from the Autonomy in Old Age project (Soch, Richter, Schütze, Kizilirmak, Assmann, Behnisch, et al., 2021; Soch, Richter, Schütze, Kizilirmak, Assmann, Knopf, et al., 2021).

## 2 MATERIALS AND METHODS

The study was approved by the Ethics Committee of the Otto‐von‐Guericke‐University Magdeburg, Faculty of Medicine, and was conducted in accordance with the Declaration of Helsinki (World Medical Association, 2013).

### 2.1 Participants

The study cohort consists of 217 neurologically and psychiatrically healthy adults. Study participants were mainly recruited by newspaper advertisements (primarily older adults) and via billboards and mailing lists at the local institutions of higher education in Magdeburg (Otto von Guericke University, University of Applied Sciences) (young adults) as well as public outreach events like the Long Night of Sciences (young and older adults). We excluded participants with insulin‐dependent diabetes (only one older adult reported taking oral antidiabetic medication) and with present depression or history of recurrent major depression, bipolar disorder, or schizophrenia. All participants gave written informed consent prior to their assessment. The sample investigated here consisted of 106 young (47 male, 59 female, age range 18–35 years, mean age 24.12 ± 4.00 years) and 111 older (46 male, 65 female, age range 60–80 years, mean age 67.28 ± 4.65 years) participants, all right‐handed by self‐report. No participant reported the use of neurological or psychiatric medication. To exclude participants with present or past psychiatric illness, alcohol, or drug dependence, the Mini‐International Neuropsychiatric Interview (M.I.N.I.; Sheehan et al., 1998; German version by Ackenheil et al., 1999) was conducted. We further assessed use of medication aside from neurological/psychiatric drugs. As expected, age‐related differences were observed. Observed differences in education levels were most likely due to historically founded differences in the education system in East Germany before and after 1989. Importantly, despite overall lower formal education level in older adults, they scored significantly higher in a German vocabulary test (“Mehrfachwahl‐Wortschatz‐Intelligenztest”; Lehrl, 2005; please see Soch, Richter, Schütze, Kizilirmak, Assmann, Behnisch, et al., 2021, supplementary discussion). A comprehensive list of the assessed characteristics for both age groups can be found in Table 1 [a more extensive description of demographics can be found in Soch, Richter, Schütze, Kizilirmak, Assmann, Behnisch, et al. (2021)].

**TABLE 1:** Demographics of young and older subjects.

	Young subjects	Older subjects	Statistics
*N*	106	111	–
Age range	18–35 years	60–80 years	–
Mean age ± SD	24.12 ± 4.00 years	67.28 ± 4.65 years	*t* = −73.11, *p* < .001
Sex ratio	47/59 m/f	46/65 m/f	χ^2^ = 0.19, *p* = .666
Ethnic composition	104/2 European/other	111/0 European/other	χ^2^ = 2.11, *p* = .146
Educational status	100/6 with/without Abitur	56/55 with/without Abitur	χ^2^ = 51.68, *p* < .001
Mean MMSE performance ± SD	–	28.84 ± 1.02 (range: 26–30)	–
MWT‐B hits ± SD	26.81 ± 3.14	30.60 ± 3.00	*z* = −8.15, *p* < .001

*Note*: Demographic information for the two age groups, along with statistics from a two‐sample *t*‐test (mean age), chi‐squared tests (sex ratio and ethnic composition), and a Mann–Whitney U‐test (MWT‐B hits). “Abitur” is the German equivalent of a high school graduation certificate qualifying for academic education.

Abbreviations: f, female; m, male; MMSE, Mini‐Mental State Examination (Creavin et al., 2016; Folstein et al., 1975); MWT‐B, multiple‐choice vocabulary test; N, sample size; SD, standard deviation.

### 2.2 Stimuli, task, and procedure of the subsequent memory paradigm

**FIGURE 2:**
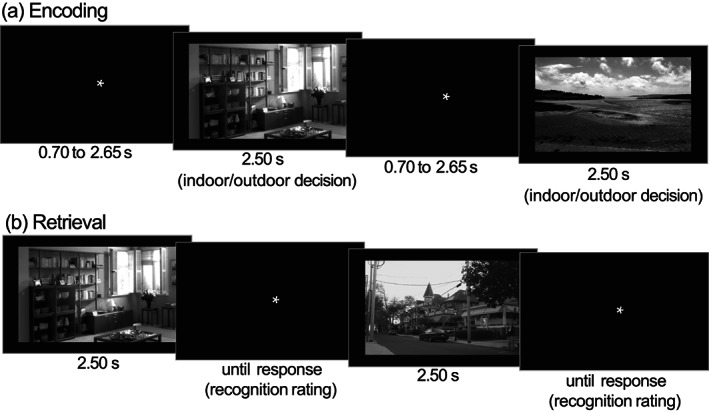
Exemplary trials of the subsequent memory task. (a) The incidental encoding task during fMRI scanning required participants to perform an indoor/outdoor decision via button press. Trial timings were independent of button press. (b) Recognition memory test, during which participants provided oral recognition ratings (5‐point scale: “sure new” over “undecided” to “sure old”) that were logged via the experimenter.

Participants performed an incidental memory encoding task undergoing fMRI scanning at 3 T. They were presented with images of various scenes and instructed to perform an indoor/outdoor rating, unaware of an upcoming surprise subsequent memory test. Exemplary trials of the encoding and memory test phases are presented in Figure 2. A total of 412 indoor and 423 outdoor images were used. The images were 8‐bit grayscale photographs of real‐world scenes, scaled to 1250 × 750 pixels resolution, and were matched for luminance (mean grey value = 127, SD = 75, units in 8‐bit RGB range of 0–255). During encoding, 90 unique pictures were presented. They comprised 44 indoor and 44 outdoor scenes presented once (hence termed *novel* images), as well as one additional indoor and one outdoor image shown 22 times each (hence termed *master* images). In total, 132 trials were presented during encoding. The highly familiarized *master* images were shown five times each for 3 s during a pre‐experimental familiarization procedure and served as a baseline for fMRI novelty contrasts (comparison of *novel* images vs. *master* images). Each encoding trial proper started with a white fixation cross on a black background (jittered from 0.70 to 2.65 s, optimized for an efficient estimation of the trial‐specific BOLD responses; Düzel et al., 2011; Hinrichs et al., 2000). The presentation of the scene image followed for 2.5 s, during which participants were instructed to perform an indoor/outdoor decision via button press. During encoding, participants were unaware that their memory of those photos would be tested later (i.e., incidental learning).

A surprise recognition memory test followed approximately 70 min (70.23 ± 3.77 min) after the start of the encoding session (i.e., about 60 min after the end of the encoding session). During this session, participants were presented with all 90 studied images (88 previously *novel* images and the 2 *master* images) and 44 new images (22 indoor/outdoor each). Upon each scene presentation, participants were requested to provide a combined recognition‐confidence rating on a 5‐point scale:

1. *Sure new*: I am sure that this picture is new.
2. *Probably new*: I think that this picture is new.
3. *Undecided*: I cannot decide if this picture is new or old.
4. *Probably old*: I think I saw this picture before.
5. *Sure old*: I am sure that I did see this picture before.

The decision was reported verbally and recorded via button press by the experimenter. A different subset of images from the pool of 835 pictures was chosen for each participant to avoid any confounding effects from individual images' memorability.

### 2.3 MRI data acquisition

Structural and functional MRI data were acquired on two Siemens 3 T MR scanners (Siemens Verio: 58 young, 64 older participants; Siemens Skyra: 48 young, 47 older participants), following the exact same protocol used in the DELCODE study (Düzel et al., 2018; Jessen et al., 2018).

First, a T1‐weighted magnetization‐prepared rapid gradient echo (MPRAGE) image (TR = 2.5 s, TE = 4.37 ms, flip‐α = 7°; 192 slices, 256 × 256 in‐plane resolution, voxel size = 1 × 1 × 1 mm) was acquired. This MPRAGE was used for regional grey matter assessment via voxel‐based morphometry (VBM), hippocampal segmentation using FreeSurfer, and optimized normalization of the functional images (see below). Moreover, a coronal T2‐weighted image was acquired perpendicular to the hippocampal axis (TR = 3.5 s, TE = 0.354 s; 64 slices, 384 × 384 in‐plane resolution, voxel size = 0.5 × 0.5 × 1.5 mm), to improve hippocampal segmentation. The hippocampal volume segmentation was performed based on both the MPRAGE and the T2‐weighted image.

The MPRAGE was followed by a 7:54 min resting‐state fMRI (rs‐fMRI) run, during which T2*‐weighted echo‐planar images (EPI; TR = 2.58 s, TE = 30 ms, flip‐α = 80°; 47 axial slices, 64 × 64 in‐plane resolution, voxel size = 3.5 × 3.5 × 3.5 mm) were acquired in odd‐even interleaved‐ascending slice order. For rs‐fMRI, participants were instructed to lie inside the scanner with eyes closed, but without falling asleep. Directly after, phase and magnitude field map images were acquired to improve correction for artifacts resulting from magnetic field inhomogeneities (unwarping, see below). This was followed by brief co‐planar T1‐weighted inversion recovery EPIs (IR‐EPI). Then, task‐based fMRI was assessed while participants performed the encoding phase of the subsequent memory paradigm (duration = 9:01 min; 206 EPI scans) using the same scanning parameters as for rs‐fMRI. Afterward, T2‐weighted FLAIR images were acquired (192 sagittal slices, TR = 5.0 s, TE = 395 ms, 256 × 256 mm in‐plane resolution, voxel size = 1.0 × 1.0 × 1.0 mm).

The complete study protocol also includes additional scanning sequences not used in the analyses reported here (fast low‐angle shot, susceptibility‐weighted imaging).

### 2.4 MRI data analysis

#### 2.4.1 Voxel‐based morphometry

For the measurement of voxel‐wise GMV and total intracranial volume (TIV), we employed voxel‐based morphometry (VBM), as implemented in the Computational Anatomy Toolbox (CAT12; http://www.neuro.uni-jena.de/cat/; Gaser & Dahnke, 2016), following a previously described protocol (Assmann et al., 2021; Richter et al., 2023). Briefly, MPRAGE images were segmented into grey matter (GM), white matter, and cerebrospinal fluid using the segmentation algorithm implemented CAT12. Segmented GM images were normalized to the Montreal Neurological Institute (MNI) reference frame, using the SPM12 DARTEL template, employing a Jacobian modulation, and keeping the spatial resolution 1 mm^3^ isotropic. The Jacobian modulation was applied to preserve voxel‐wise information on local tissue volume. We, therefore, refer to grey matter volume (GMV). Normalized GM maps were smoothed with a Gaussian kernel of 6 mm at FWHM.

The FLAIR images were used to assess white matter lesion volume (WMLV) via automatic segmentation using the Lesion Prediction Algorithm (Schmidt, 2017), as implemented in the Lesion Segmentation Toolbox (LST v3.0.0; https://www.applied-statistics.de/lst.html) based on CAT12. Older adults typically show a higher amount of small WM lesions, mostly of vascular origin which was also the case in our sample (see Supplementary Results 2.1), and we accounted for this in our models by including WMLV as a covariate.

#### 2.4.2 Hippocampus segmentation

Hippocampal volumes were calculated based on the automated segmentation using FreeSurfer 6.0 (Fischl, 2012; https://surfer.nmr.mgh.harvard.edu/) and the module for segmentation of hippocampal subfields (Iglesias et al., 2015; Quattrini et al., 2020). In addition to the MPRAGE images, high‐resolution T2‐weighted images acquired perpendicular to the hippocampal axis (see Section 2.3) were included in the FreeSurfer pipeline, to improve segmentation (Dounavi et al., 2020).

#### 2.4.3 Preprocessing of fMRI data (rest and task)

All preprocessing was carried out using Statistical Parametric Mapping version 12 (SPM12; Wellcome Trust Center for Neuroimaging, University College London, London, UK). Both task‐fMRI and rs‐fMRI data were preprocessed with the same protocol to ensure comparability,^1^ and were identical to those used in our earlier works (Soch, Richter, Schütze, Kizilirmak, Assmann, Behnisch, et al., 2021; Soch, Richter, Schütze, Kizilirmak, Assmann, Knopf, et al., 2021). EPIs were corrected for acquisition time delay (slice timing), head motion (realignment), and magnetic field inhomogeneities (unwarping), using voxel‐displacement maps derived from the field maps. The MPRAGE image was spatially co‐registered to the mean unwrapped image and segmented into six tissue types, using the unified segmentation and normalization algorithm implemented in SPM12. The resulting forward deformation parameters were used to normalize unwrapped EPIs into a standard stereotactic reference frame (Montreal Neurological Institute, MNI; voxel size = 3 × 3 × 3 mm). Normalized images were spatially smoothed using an isotropic Gaussian kernel of 6 mm full width at half maximum.

#### 2.4.4 Preparation of rs‐fMRI data

PerAF was computed from the preprocessed rs‐fMRI images using an adapted version^2^ of the RESTplus toolbox (Jia et al., 2019). PerAF is a scale‐independent measure of the percentage of BOLD fluctuations relative to the mean BOLD signal intensity for each time point, which has been averaged across the whole time series (Jia et al., 2020). It is computed for BOLD variations in the range of 0.01–0.08 Hz as follows:

$$\text{PerAF}=\frac{1}{n}\sum_{i=1}^{n}\left|\frac{y_{i}-\mu }{\mu }\right|\cdot 100$$

where $y_{i}$ is the signal intensity of the ith time point, n is the total number of time points of the time series and μ is the mean value of the time series [see Jia et al. (2020) for a comparison of different rs‐fMRI measures like amplitude of low‐frequency fluctuation (ALFF) and fractional ALFF]. For our present analyses, we used mean PerAF (mPerAF, i.e., the ratio of a given voxel's PerAF and the global mean). A previous study suggests that mPerAF outperforms other common rs‐fMRI amplitude measures with regard to test–retest reliability and is a useful analog to the percent signal change used in task‐based fMRI (Jia et al., 2020).

#### 2.4.5 First‐level modeling of task‐based fMRI data

For task‐based fMRI, we were interested in the subsequent‐memory effect. To this end, we computed a general linear model (GLM) on the fMRI data acquired during the encoding session of the subsequent memory paradigm. We implemented two onset regressors, one for the to‐be‐encoded images (novelty regressor) and one for presentations of the two over‐familiarized images (master regressor). Both regressors were created as short box‐car stimulus functions with an event duration of 2.5 s, convolved with the canonical hemodynamic response function, as implemented in SPM12. The regressor reflecting subsequent‐memory performance was obtained by parametrically modulating the novelty regressor with a function describing the responses in the delayed recognition memory test. The parametric modulation employed an arcsine transformation, which puts a higher weight on items that received a “sure old” or “sure new” response compared with items where participants were less sure (Soch, Richter, Schütze, Kizilirmak, Assmann, Knopf, et al., 2021, Figure 2a). Specifically, the parametric modulator (PM) was given by

$$\mathrm{PM}=\arcsin\left(\frac{x-3}{2}\right)\cdot \frac{2}{\pi }$$

where $x\in \left\{1,2,3,4,5\right\}$ is the response rating from the subsequent memory test as described under Section 2.2, such that –1≤PM≤+1. The model further included the six rigid‐body movement parameters obtained from realignment as covariates of no interest, and a constant regressor representing the implicit baseline. A non‐negative effect of the parametric modulator reflects differential activation for remembered versus forgotten items and is referred to as the subsequent memory effect (SME) in the following and has to be interpreted as follows:

- Positive contrast estimates reflect relative activations associated with later remembering.
- Negative contrast estimates reflect relative activations associated with later forgetting.
- Contrast estimates around zero reflect no association between observed fMRI activity and later remembering or forgetting.

In other words, the term SME denotes a relationship of the stimulus‐specific BOLD response with later memory, but the sign is relevant for knowing whether it is related to remembering or forgetting.

#### 2.4.6 Statistical modeling

All hypotheses were tested using GLMs. We employed a newly developed MATLAB‐based toolbox^3^ for multi‐modal neuroimaging analyses, in combination with SPM12 running on MATLAB R2019b. Voxel‐wise, multi‐modal^4^ analyses were necessary to test the relationship between resting‐state and encoding‐related activity, that is, between mPerAF and SME. Moreover, multi‐modal analyses also allowed taking voxel‐wise confounding factors such as regional differences in GMV into account.

To assess activation in the DMN, we used masks of functionally defined regions of interest (ROIs) from an independent study (Shirer et al., 2012). This set of ROIs consists of 10 dorsal and 9 ventral DMN ROIs depicted in 3. We employed both a composite ROI covering the entire DMN and individual ROIs of the mPFC/rACC, PCC, and precuneus.

As participants were scanned with two different scanners, including different head coils, we needed to assess the potential influence of scanner on all imaging variables (mPerAF, SME, and GMV). Additionally, the influence of sex was assessed. Depending on the results, we would include the respective variable(s) as covariate(s) in the models of interest to test the hypotheses.

All MATLAB scripts for the models are provided at OSF^5^ and the MRI data at NeuroVault^6^. All results are reported FWE‐corrected with *p* < .05, cluster threshold = 10, if not stated otherwise. An inclusive mask of active voxels for mPerAF and SME was used.

To assess potential confounding effects of sex and scanner, we ran the following analyses:

- mPerAF ~ scanner(Skyra, Verio) + sex(m,f)
- SME ~ scanner(Skyra, Verio) + sex(m,f)
- GMV ~ scanner(Skyra, Verio) + sex(m,f) + age + TIV

with scanner and sex as categorical and age and TIV as continuous regressors.

We expected no effects of scanner or sex on mPerAF or SME. Furthermore, we expected no effect of scanner on GMV, and including TIV as a covariate should also control for possible effects of sex on GMV. Age was included in the model for GMV out of interest, because GMV was not assessed as a dependent variable in the main analyses (models 1–3), where age was a main independent variable of interest. For clarity, GLMs addressing effects of potential confounding factors are reported in the supplement in detail (see Supplementary Results 2.2), but we provide a brief overview below.

First, we assessed potential influences of sex and scanner on mPerAF (see A). To this end, we included both as categorical factors (i.e., a 2 × 2 design with four regressors: male_Skyra, male_Verio, female_Skyra, female_Verio). We observed a main effect of scanner, but no main effect of sex, nor an interaction. For detailed descriptions of the effect of scanner, see Supplementary Results 2.2, Figure S2a, and Table S3. Second, we tested potential influences of sex and scanner on the fMRI correlates of successful memory encoding (see B). We found no effect of sex or scanner, nor an interaction. Third, we tested the influence of sex, scanner, age, and TIV on GMV (see C). We found no main effects of scanner or sex, nor an interaction, but, as to be expected, main effects of age and TIV (see Figure S3 and Table S4).

To summarize, it is necessary to include *scanner* in models including mPerAF. For the comparison between activity during rest (i.e., mPerAF) and memory encoding, the same was done for SME models. Scanner was included as a categorical factor. It did not seem necessary to include *sex* in any model including only mPerAF or SME fMRI data. Considering the significant effect of TIV on GMV observed here, we included TIV as a continuous covariate in all analyses including GMV.

The first hypothesis, stating that BOLD signal variation within the DMN, and especially mPFC and precuneus *at rest* as assessed by rs‐fMRI mPerAF is independent of HC volumes, was tested using the following model^7^:

(1) $$\begin{array}{l} \text{mPerAF}\sim \mathrm{age}\_\text{group}\left(\text{young,}\mathrm{old}\right)+\text{scanner(Verio, Skyra)}\\ \;+\;\mathrm{HC}\_\mathrm{vol}+\mathrm{TIV}+\text{WMLV}+\mathrm{age}+\mathrm{GMV} \end{array}$$

with age‐group‐wise and scanner‐wise GMV as a voxel‐wise imaging covariate to account for regional GMV differences and age‐group‐wise HC volume, TIV,^8^ and WMLV, and (collapsed across age‐groups) age as non‐imaging covariates. All continuous covariates were age‐group‐wise mean‐centered. Note that the covariate *age* only contained the residual age‐variance within each age group. The following regressors were included into the model: young_Skyra, young_Verio, older_Skyra, older_Verio, HCvol_young, HCvol_old, TIV_young, TIV_old, WMLV_young, WMLV_old, age, GMV_young_Skyra, GMV_young_Verio, GMV_old_Skyra, and GMV_old_Verio. An inclusive mask of active voxels for mPerAF and SME was used, which essentially comprised the whole brain.

The second hypothesis, stating that DMN hyperactivation (or rather reduced deactivation) during successful encoding is negatively related to hippocampal volumes in older adults, was tested with the following model:

(2) $$\begin{array}{l} \mathrm{SME}\sim \mathrm{age}\_\text{group}\left(\text{young,}\mathrm{old}\right)+\text{scanner(Verio, Skyra)}\\ \;+\;\mathrm{HC}\_\mathrm{vol}+\mathrm{TIV}+\text{WMLV}+\mathrm{age}+\mathrm{GMV} \end{array}$$

where HC volume, TIV, WLMV, age, and GMV were age‐group‐wise mean‐centered (i.e., the same regressors as in model 1). Again, scanner was included, because we needed fully comparable models to compare the effects of HC volume. Hence, the following regressors were included in the model: young_Skyra, young_Verio, older_Skyra, older_Verio, HCvol_young, HCvol_old, TIV_young, TIV_old, WMLV_young, WMLV_old, age, GMV_young_Skyra, GMV_young_Verio, GMV_old_Skyra, and GMV_old_Verio. An inclusive mask of active voxels for mPerAF and SME was used.

**FIGURE 3:**
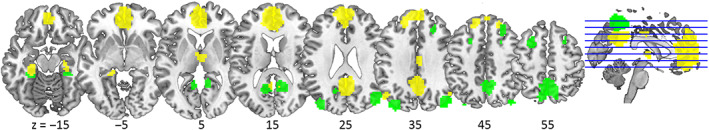
ROI clusters for dorsal (green) and ventral (yellow) DMN. These functional ROIs were taken from Shirer et al. (2012) and combined for the present analyses.

The third hypothesis that DMN hyperactivation during successful encoding is independent of the degree of possible general DMN hyperactivation at rest, was tested with:

(3) $$\begin{array}{l} \mathrm{SME}\sim \mathrm{age}\_\text{group}\left(\text{young,}\mathrm{old}\right)+\text{scanner(Skyra, Verio)}+\mathrm{age}\\ \;+\;\text{mPerAF}+\mathrm{GMV} \end{array}$$

where age was mean‐centered by age group, and mPerAF was included as a mean‐centered imaging covariate. The model had the following regressors: young_Skyra, young_Verio, older_Skyra, older_Verio, age, mPerAF_young_Skyra, mPerAF_young_Verio, mPerAF_old_Skyra, mPerAF_old_Verio, GMV_young_Skyra, GMV_young_Verio, GMV_old_Skyra, and GMV_old_Verio. An inclusive DMN mask as described above was applied (Figure 3).

#### 2.4.7 Exploratory statistical analyses

Analyses to compare behavioral memory performance and hippocampal volumes between age groups were conducted in R version 4.1.2 (R Core Team, 2021) and RStudio version 2021.9.1.372 (RStudio Team, 2021). Boxplots were also generated using R. Additional R packages used for computing inferential statistics were ggstatsplot (Patil, 2021) and pander. When reporting results of analyses of covariances, we tested via Levene's test whether equal variances could be assumed. As this was the case for all, no adjustments were needed.

In addition to the analyses described in our initial analysis plan, we computed a set of exploratory statistics to further characterize the relationship of DMN activity at rest and during encoding with hippocampal volumes as well as behavioral memory performance. To enable statements not only about the evidence of the presence of an effect, but also its absence, we performed Bayesian correlational analyses (Pearson correlation) between three memory measures, hippocampal volumes, and ROI‐wise mean contrast estimates for SME and resting‐state mPerAF. This was done because we had observed null effects for hippocampal volumes and DMN activity. BFs of 1 can be interpreted as no evidence for either the null (H0) or the alternative hypothesis (H1). BF_10_ denotes evidence for H1 and BF_01_ for H0, whereby BF_01_ = 1/BF_10_. A BF_10_ between 1 and 3 can be interpreted as anecdotal evidence for H1, 3–10 as moderate evidence, and >10 as strong evidence (Schönbrodt & Wagenmakers, 2018). Bayesian analyses were conducted using JASP version 0.16.02 (JASP Team, 2022).

## 3 RESULTS

### 3.1 Hippocampal volumes

**FIGURE 4:**
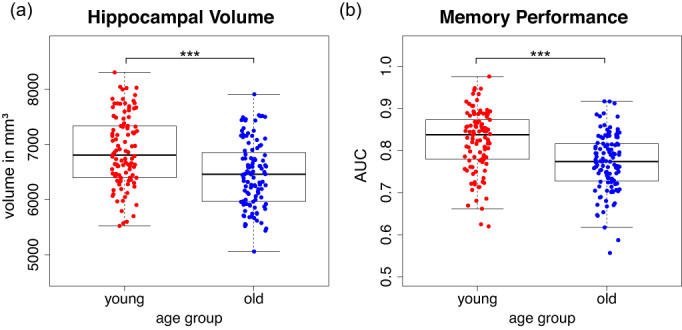
Age group differences regarding total hippocampal volume (panel a) and memory performance (area under the curve “AUC”, panel b). The box length represents the interquartile range (IQR), and the ends of the whiskers are at ±1.5 IQR, respectively. The bold black bars represent the median.

To assess potential age‐related differences in hippocampal (HC) volumes, we compared total HC volumes, as well as input and output regions of the HC (see Supplementary Results 2.2 and Table S2). Hippocampal volumes differed significantly between age groups as assessed by an ANCOVA [effect of age group on HC volume: *F*(1,214) = 29.92, *p* < .001], including TIV as a covariate [effect of TIV: *F*(1,214) = 20.70, *p* < .001]. Older adults showed significantly lower HC volume (mean = 6453.07 mm^3^, SD = 593.21 mm^3^) compared with young adults (mean = 6890.91 mm^3^, SD = 638.65 mm^3^; Figure 4a).

### 3.2 Behavioral memory performance

Memory responses were recorded on a 5‐point recognition‐confidence rating scale (see Section 2.2). Therefore, there were no classical binary hit and false alarm rates, but memory responses as a function of this rating scale. We thus quantified memory performance as the area under the curve (AUC) when plotting the proportion of responses for hits (= recognition‐confidence rating for truly old items) against that of false alarms (= recognition‐confidence rating for new items; but see figure 8 and appendix B in Soch, Richter, Schütze, Kizilirmak, Assmann, Behnisch, et al., 2021).

Older adults (mean = 0.77, SD = 0.07) had significantly lower AUCs compared to young adults (mean = 0.82, SD = 0.07; see Figure 4b) as tested via an independent‐samples *t*‐test [*t*(215) = 5.40, *p* < .001]. Thus, older adults showed poorer discriminative memory performance. The relationship between memory performance and hippocampal volume is reported in Section 3.3.4.

### 3.3 Assessment of age‐related functional involvement of DMN structures at rest and during successful encoding

#### 3.3.1 Default mode network activity at rest is modulated by age, but not hippocampal volume

**FIGURE 5:**
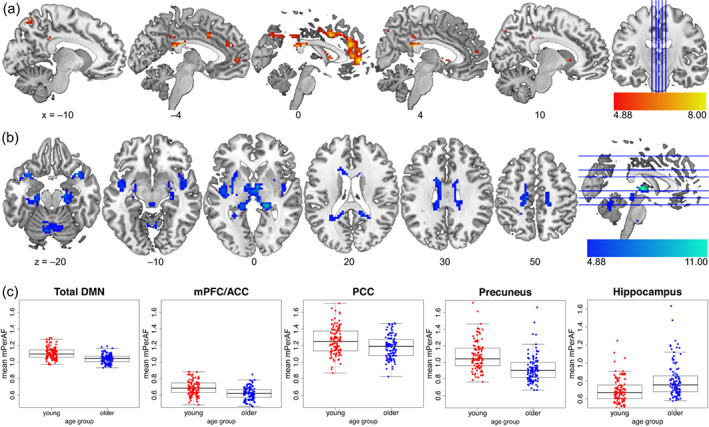
Age group differences in resting‐state mPerAF. The young > older contrast is shown in (a), older > young in (b), and (c) depicts mean ROI estimates for functional ROIs from the DMN ROI set by Shirer et al. (2012), plus a right hippocampal ROI (4 mm radius sphere around peak voxel [36, –13, –22] for the older>young contrast), illustrating our unexpected finding of higher hippocampal mPerAF in older adults. The lower ends of the scales reflect the FWE‐corrected significance thresholds.

Age group comparisons showed that, across the whole brain (Table 2), younger adults showed higher mPerAF in medial parts of the DMN (Figure 5a), while older adults showed higher BOLD amplitude fluctuations at rest (mPerAF) in cerebral white matter (Figure 5b), and, importantly in MTL regions, that is, in the bilateral parahippocampal cortices and right hippocampus, while. Generally, younger adults showed higher amplitude fluctuations within the DMN compared to older adults, and this difference was especially pronounced within the precuneus (Figure 5c).

**TABLE 2:** Effects of age group on mPerAF.

Anatomical label	Cluster size	Peak *p* (FWE‐corr)	Peak *T*	*x*, *y*, *z* (mm)
*Young > older*				
Anterior cingulate cortex	162	<.001	8.07	0, 53, 2
Anterior cingulate cortex		<.001	7.48	0, 41, 23
Middle cingulate and paracingulate gyri		<.001	7.15	0, 14, 38
	39	<.001	7.66	3, –37, 23
		<.001	7.06	−3, −25, 26
L superior temporal gyrus	11	<.001	7.44	−48, 17, –10
R inferior parietal gyrus	28	<.001	6.60	42, –40, 56
R postcentral gyrus		.001	5.66	51, –25, 53
R inferior frontal gyrus	41	<.001	6.35	54, 14, 29
R middle frontal gyrus		<.001	5.99	45 20 41
L precuneus	20	<.001	6.30	−9, −67, 59
L superior parietal gyrus		.003	5.49	−21, −67, 59
L middle temporal gyrus	25	<.001	6.21	−39, −52, 20
L angular gyrus		.001	5.68	−42, −52, 29
L inferior parietal gyrus		.002	5.61	−48, −52, 38
	19	<.001	6.11	0, 11, 5
R caudate nucleus		.001	5.81	9, 14, 2
R caudate nucleus		.002	5.59	18, 23, 2
R middle cingulate and paracingulate gyri	30	<.001	6.02	3, –37, 38
L middle cingulate and paracingulate gyri		.003	5.51	−9, −40, 32
R precuneus	28	.001	5.80	3, –73, 41
precuneus		.003	5.49	0, –64, 38
R precentral gyrus	12	.003	5.49	21, –22, 71
*Older > young*				
	680	<.001	11.59	0, –13, 2
R thalamus: pulvinar anterior		<.001	9.71	18, –31, −1
		<.001	9.60	−12, −34, 2
	44	<.001	8.15	18, –16, −19
R parahippocampal gyrus		<.001	7.17	21, –25, −19
R hippocampus		.005	5.39	36, –13, –22
L Heschl's gyrus	202	<.001	8.12	−42, −19, 8
L superior temporal gyrus		<.001	7.77	−33, 14, –19
L insula		<.001	7.31	−45, −10, 2
R insula	159	<.001	8.05	36, 14, –16
R insula		<.001	7.54	42, –16, 5
R insula		<.001	6.48	45, 5, –10
	119	<.001	7.85	9, –28, −43
		<.001	7.35	−9, −25, −40
L parahippocampal gyrus		<.001	6.90	−18, −19, −19
	277	<.001	7.59	−24, −28, 47
		<.001	7.12	−18, −40, 26
		<.001	7.12	−15, −22, 50
L lobule VI of cerebellar hemisphere	246	<.001	7.49	−6, −67, −16
L lobule VI of cerebellar hemisphere		<.001	6.56	−15, −64, −22
L lobule IV, V of cerebellar hemisphere		<.001	6.30	−18, −52, −25
R supplementary motor area	46	<.001	6.60	15, –28, 53
		<.001	6.45	21, –25, 47
		.001	5.82	18, –16, 50
	10	<.001	6.26	12, 14, 23

*Note*: Table shows three local maxima more than 8.0 mm apart. Anatomical labels for coordinates according to AAL3 atlas (Rolls et al., 2020). Height threshold: *T* = 4.88; extent threshold: *k* = 10 voxels.

Positive and negative effects of HC volume on mPerAF within the DMN were assessed separately for each age group. There was no effect of HC volume on mPerAF, neither across the cohort nor for either age group separately. Considering the volumes of hippocampal input and output regions separately yielded the same outcome (see Supplementary Results, 2.4).

It should be noted that GMV as a voxel‐wise imaging covariate had effects on mPerAF within distributed white matter regions (see Supplementary Results, Figure S5a and Table S6), which supports the importance of including this confounding factor in the analysis.

#### 3.3.2 DMN activity during memory encoding is age‐dependent, but independent of hippocampal volumes

**FIGURE 6:**
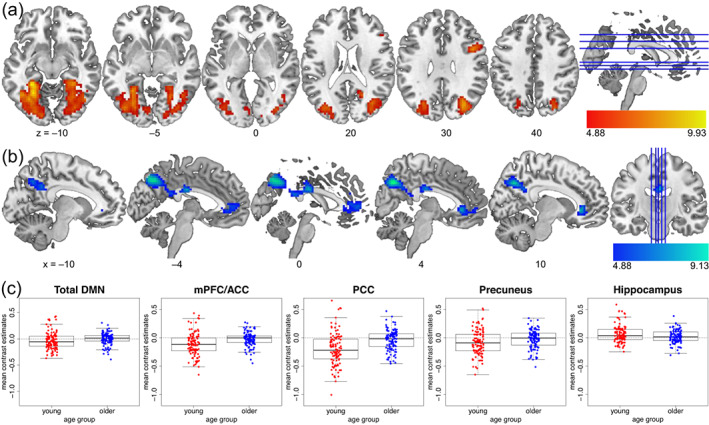
Age group differences for the subsequent memory effect. The young > older contrast is shown in (a), older > young in (b), and (c) depicts mean contrast estimates for the respective ROIs as in Figure 5c for comparability, that is, for functional ROIs from the DMN ROI set by Shirer et al. (2012), plus the same hippocampal ROI (4 mm radius sphere around peak voxel [36, –13, –22]). The lower ends of the color bars reflect the FWE‐corrected significance thresholds.

When assessing general effects of age group on memory encoding (Table 3), we found that younger participants showed relatively higher values in visual processing regions than older adults (lingual gyrus, occipital gyrus; Figure 6a, but see Figure S5 for age‐group‐wise plots of the positive effect of the parametric modulator). In contrast, older adults showed lower deactivations within DMN midline regions (precuneus, PCC, ACC, mPFC; Figure 6b) compared to young adults (but see Figure S6). In line with our parametric SME model, older adults further show a reduced relationship between activity within DMN regions and memory formation (Figure 6c), because their values were closer to zero (but see explanation at the end of Section 2.4.5).

**TABLE 3:** Effects of age group on SME.

Anatomical label	Cluster size	Peak *p* (FWE‐corr)	Peak T	*x*, *y*, *z* (mm)
*Young > older*				
L fusiform gyrus	977	<.001	9.97	−27, −52, −7
L fusiform gyrus		<.001	9.64	−30, −43, −10
L fusiform gyrus		<.001	8.01	−33, −64, −10
R fusiform gyrus	986	<.001	8.29	27, –37, −13
R middle occipital gyrus		<.001	8.28	30, –76, 35
R middle occipital gyrus		<.001	8.18	30, –67, 32
R inferior frontal gyrus	72	<.001	7.61	45, 8, 29
R cuneus	62	<.001	6.70	21, –58, 20
R calcarine fissure and surrounding cortex		<.001	6.64	18, –52, 8
R inferior frontal gyrus, triangular part	37	<.001	6.28	45, 32, 14
	13	<.001	6.07	−18, −58, 14
*Older > young*				
R precuneus	499	<.001	9.16	6, –64, 38
L precuneus		<.001	8.55	−3, −67, 41
R middle cingulate and paracingulate gyri		<.001	7.81	3, –19, 32
R anterior cingulate cortex, subgenual	164	<.001	7.81	9, 32, –1
L anterior cingulate cortex, pregenual		<.001	6.44	0, 53, 5
L anterior cingulate cortex, pregenual		<.001	6.09	0, 41, 8
R angular gyrus	57	<.001	7.02	54, –52, 32
R superior frontal gyrus. dorsolateral	11	0.001	5.66	21, 56, 26
R middle frontal gyrus		0.004	5.41	27, 50, 32
L inferior parietal gyrus	30	0.001	5.65	−45, −52, 38
L angular gyrus		0.013	5.17	−51, −55, 29

*Note*: Table shows three local maxima more than 8.0 mm apart. Anatomical labels for coordinates according to AAL3 atlas. Height threshold: *T* = 4.88, extent threshold: *k* = 10 voxels.

**FIGURE 7:**
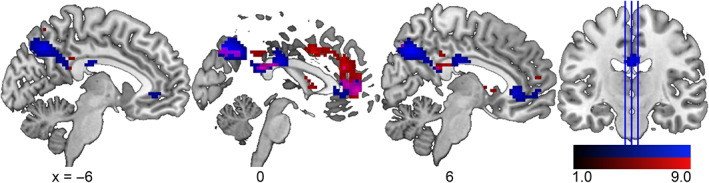
Comparison of age group differences at rest and during memory encoding. Shown is an overlap (magenta) between age group differences for young > older resting‐state amplitude of fluctuations (red, Figure 5b) and older > young subsequent memory effect (blue, Figure 6a). The lower ends of the scales do not reflect the significance thresholds but have been chosen to enhance the visibility of the overlap in magenta.

The age‐group difference of SME maps (older > young) showed an anatomically similar pattern to that of resting‐state mPerAF (Figures 5a and 6b), with reduced encoding‐related deactivations in regions that showed lower BOLD amplitude fluctuations at rest. In an exploratory analysis, we determined the exact overlap (Figure 7) by inclusively masking the two contrasts. Although the intersection was relatively small, it confirmed the visual impression that the overlap of these two whole‐brain contrast images was in midline regions of the DMN (Table 4).

**TABLE 4:** Overlap between age group differences for mPerAF young > old and SME old > young.

Anatomical label	Cluster size	Peak *p* (FWE‐corr)	Peak T	*x*, *y*, *z* (mm)
R precuneus	27	<.001	8.92	3, –67, 41
	29	<.001	7.01	−3, −22, 29
		<.001	6.45	0, –40, 23
L anterior cingulate cortex, pregenual	38	<.001	6.44	0, 53, 5
L anterior cingulate cortex, pregenual		<.001	6.09	0, 41, 8
L angular gyrus	10	.002	5.53	−45, −52, 32

There was no effect of HC volume on SME in either older or young adults. When assessing the effects of the hippocampal input and output region volumes separately, we also found no effects surviving the significance threshold (Supplementary Results 2.6).

Here, GMV as a potential confounding factor only had a small impact on SME, restricted to a small cluster in the left precuneus (see Figure S5B and Table S6).

To summarize, while we observed pronounced age‐group effects, our hypothesis regarding hippocampal volumes (Hypothesis 2) was not supported by the data, with DMN activity during successful memory encoding being unaffected by HC volume.

#### 3.3.3 Encoding‐related activity in DMN midline regions is negatively related to resting‐state DMN amplitude in young adults

**FIGURE 8:**
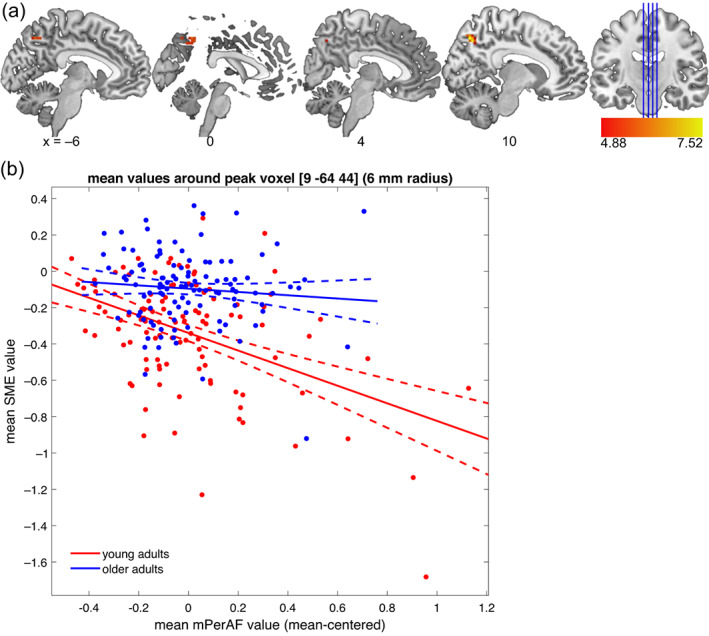
Negative effect of resting‐state mPerAF on SME. (a) Effect in young adults. An inclusive DMN mask was used to assess the relationship between resting‐state amplitude fluctuations and task‐fMRI subsequent memory effect. (b) Scatter plot for mean contrast estimates within a 6‐mm radius sphere around the peak voxel of the negative effect in young adults. The negative effect did not reach significance in older adults. As can be seen, no positive effect was observed in either group.

Contrary to our hypothesis, we found that resting‐state amplitude (mPerAF) was indeed associated with memory‐encoding‐related activity within the DMN (Hypothesis 3). More specifically, we observed a negative effect of resting‐state mPerAF on SME in young, but not older, adults in clusters within bilateral precuneus (see Figure 8 and Table 5). As can be gathered from Figure 8b, the effect was reflected by higher successful encoding‐related deactivations within the precuneus going along with higher resting‐state amplitudes during rest.

**TABLE 5:** Effects of resting‐state mPerAF on SME (inclusive DMN mask).

Anatomical label	Cluster size	Peak *p* (FWE‐corr)	Peak T	*x*, *y*, *z* (mm)	
*Positive effect of mPerAF* (*collapsed across age groups*)					
L mid occipital gyrus	10	<.001	5.87	−33, −82, 29	
L mid occipital gyrus		.001	5.68	−39, −76, 29	
*Negative effect of mPerAF in young participants only*					
R precuneus	18	<.001	7.55	9, –67, 47	
L precuneus		.006	5.33	0, –67, 47	
L precuneus	21	<.001	6.70	−3, −61, 41	
L precuneus		<.001	5.85	0, –55, 47	

*Note*: Table shows three local maxima more than 8.0 mm apart. Anatomical labels for coordinates within grey matter according to AAL3 atlas. Height threshold: *T* = 4.88, extent threshold: *k* = 10 voxels. No negative effect collapsed across age groups was found. Neither a positive nor a negative effect of mPerAF was found in older adults.

Beyond the negative effect on encoding‐related DMN activity in young adults, mPerAF also had a positive effect on SME collapsed across age groups. However, this was restricted to a 10‐voxel cluster within middle occipital gyrus, which, while formally part of our DMN ROI, lies outside the structures of interest (midline structures, especially mPFC and precuneus). Furthermore, the effect did not survive FWE correction when tested separately within the age groups. The other imaging covariate, GMV, did not have a significant effect in this model.

#### 3.3.4 Relationship between DMN activity during successful encoding and rest with behavioral memory performance and hippocampal volume

We had hypothesized a relationship between hippocampal volume an DMN involvement during successful encoding, but no relationship between hippocampal volume and DMN involvement at rest (Figure 1), but we in fact observed null effects for hippocampal volume on both (Sections 3.3.1 and 3.3.2). To be able to not only about the presence of an association, but also about the absence of effects, we calculated Bayesian correlations between DMN ROIs and hippocampal volume as well as memory performance measures (Table 6 but see Figure S8 for scatterplots). For mPerAF, the correlations with memory performance were not part of our original analysis plan and thus exploratory.

**TABLE 6:** Correlations of mPerAF and SME with memory performance and hippocampal volumes (Bayesian Pearson correlations).

	Total DMN			mPFC/ACC			PCC			Precuneus		
	*r*	BF_10_	BF_01_	*r*	BF_10_	BF_01_	*r*	BF_10_	BF_01_	*r*	BF_10_	BF_01_
*mPerAF*												
AUC												
Young	.064	0.150	6.667	−.103	0.210	4.762	.143	0.346	2.890	.021	0.124	8.065
Older	**.283**	**10.243**	0.098	.173	0.607	1.647	.205	1.184	0.845	.056	0.140	7.143
VLMT												
Young	.214	1.176	0.850	.010	0.126	7.937	**.255**	**3.135**	0.319	.162	0.450	2.222
Older	.109	0.223	4.484	**.255**	**3.712**	0.269	.044	0.134	7.463	−.198	0.943	1.060
WMS												
Young	.121	0.255	3.922	−.036	0.132	7.576	.092	0.188	5.319	.105	0.213	4.695
Older	.115	0.239	4.184	.209	1.178	0.849	.082	0.171	5.848	−.143	0.346	2.890
HC volume												
Young	−.019	0.124	8.065	−.103	0.209	4.785	.083	0.173	5.780	.024	0.125	8.000
Older	.099	0.202	4.950	.072	0.157	6.369	.060	0.144	6.944	.072	0.157	6.369
*SME*												
AUC												
Young	.054	0.141	7.092	−.011	0.122	8.197	−.011	0.122	8.197	.076	0.163	6.135
Older	−.042	0.130	7.692	−.060	0.144	6.944	−.076	0.162	6.173	−.138	0.332	3.012
VLMT												
Young	.063	0.151	6.623	−.078	0.168	5.952	−.015	0.126	7.937	.130	0.285	3.509
Older	−.100	0.204	4.902	−.031	0.128	7.813	−.096	0.195	5.128	**−.267**	**5.265**	0.190
WMS												
Young	.081	0.171	5.848	.032	0.130	7.692	.035	0.131	7.634	−.052	0.141	7.092
Older	.107	0.219	4.566	.110	0.227	4.405	.013	0.123	8.130	.028	0.127	7.874
HC volume												
Young	−.006	0.122	8.197	−.037	0.130	7.692	−.027	0.126	7.937	.006	0.122	8.197
Older	−.013	0.120	8.333	−.016	0.120	8.333	−.053	0.138	7.246	−.006	0.119	8.403

*Note*: The table reports Pearson's *r* as well as Bayes factors for the H_1_, that is, the presence of a correlation (BF_10_), and the H_0_, that is, the absence of a correlation (BF_01_ = 1/BF_10_, but we report it explicitly to show the evidence for the absence of relationships to hippocampal volume). To highlight the most robust correlations, we have printed all *r* and BF_10_ > 3 in bold letters.

When assessing the relationship between memory performance and DMN BOLD signal changes, we identified several correlations between resting‐state mPerAF within the DMN and memory performance. In older adults, we observed strong evidence for a (positive) correlation between AUC (memory performance in the fMRI encoding task) and mean mPerAF in the whole DMN. Therefore, we computed exploratory correlations with two independent measures of memory: Verbal Learning and Memory Test (VLMT; Helmstaedter et al., 2001) and Wechsler Memory Scale (WMS; Härting et al., 2000) scores for 1‐day delayed‐recall (see Richter et al., 2023). Those tests were part of an extensive neuropsychological test battery participants underwent besides structural and functional MRI. Unlike the AUC from the fMRI *incidental* encoding task, these tests assessed memory after *intentional* encoding. In older adults, we observed moderate evidence for a positive correlation between VLMT performance with mean mPerAF in mPFC/ACC. Young adults showed a positive correlation between mean mPerAF of the PCC ROI and VLMT scores.

Second, older adults showed a negative correlation between mean SME contrast estimates within the precuneus and 1‐day delayed VLMT performance (i.e., delayed recall of word lists). Thus, higher mean values (i.e., lower deactivations or above‐baseline activations) within the precuneus during memory encoding were associated with poorer 1‐day delayed recall in an unrelated verbal memory task. Otherwise, no correlations between behavioral memory performance and SME within DMN ROIs were observed.

Congruent with the previous multimodal voxel‐wise analyses (Sections 3.3.1 and 3.3.2), there was moderate evidence for the absence of any correlation between HC volume and either resting‐state mPerAF or encoding‐related activity in all DMN ROIs.

## 4 DISCUSSION

The main goal of this study was to directly assess the relationship between DMN activity at rest and during successful long‐term memory encoding in relation to aging effects. We tested whether the relatively higher DMN activity during successful encoding in older adults reported previously (Maillet & Rajah, 2014) might be (i) a sign of compensatory, for example, more self‐ or prior knowledge‐related, hippocampus‐independent encoding strategies (Schott et al., 2019; van Kesteren et al., 2013), which can support memory formation in older age (Wynn et al., 2020), or whether (ii) it merely reflects the inability to suppress DMN activity during task execution, which is the more common interpretation (Grady et al., 2006; Hafkemeijer et al., 2012). To this end, we directly investigated the relationship between amplitude of fluctuations at rest (i.e., mPerAF) and a parametrically modeled subsequent memory effect (i.e., SME) in young and older adults, as well as the influence of hippocampal volumes (Figure 1).

While younger adults showed a negative relationship between mPerAF and SME in the precuneus, a core region of the posterior DMN, no such relationship was observed in older adults (Figure 8). Nevertheless, older adults showed both lower mPerAF within the DMN at rest (Figure 5c) and lower task‐related DMN deactivations during successful encoding (Figure 6c). Furthermore, in a set of correlational analyses between ROI‐wise mean signals and memory performance (Table 6), in older adults, higher resting‐state mPerAF within the DMN (mPFC/ACC, but not precuneus) was positively correlated with memory performance in established tests of episodic memory. Additionally, again in older adults only, there was a negative correlation between successful encoding‐related activity in the precuneus and memory performance. This may indicate the relevance of the ability to modulate functional activity for cognitive maintenance.

### 4.1 Older adults show reduced activity modulation within the DMN at rest and during successful memory encoding

In a relatively large sample of 217 healthy participants, we could replicate the previously described age group differences in fMRI correlates of episodic encoding (Maillet & Rajah, 2014), with younger adults showing higher inferior and medial temporal activity and older adults showing a lower deactivation or even activation of DMN midline structures (precuneus, PCC, rACC, and mPFC; Figure 6). In accordance with the parametric SME model employed here, this suggests that older adults generally show a lower association between DMN activity during successful memory formation. This might indicate a decoupling of DMN activity during encoding and encoding success in older adults, an interpretation that somewhat differs from the common interpretation that reduced DMN deactivation during encoding indicates a reduced ability to suppress task‐unrelated thoughts (Hayes et al., 2017; Persson et al., 2014; Sambataro et al., 2010). Our interpretation is in line with a longitudinal study showing higher variability in the organization of functional networks with increasing age (Malagurski et al., 2020).

We further found considerable age‐related differences in resting‐state mPerAF (Figure 5). Compared to young adults, older adults showed lower mPerAF within the precuneus, PCC, mPFC, and rACC, and thus in the same regions in which they showed lower deactivations during successful memory encoding. However, these reduced deactivations cannot simply be explained by the overall reduced fluctuation of DMN activity, as the negative relationship between DMN mPerAF and SME found in young adults was absent in the older age group.

In previous studies of memory encoding in older adults, both reduced activations of task‐relevant brain regions and reduced deactivations of task‐irrelevant brain regions (i.e., mainly the DMN) have been associated with poorer memory performance in older compared to younger adults (Davis et al., 2008; Dennis et al., 2008; Miller et al., 2008). The deviation of an individual's encoding‐related brain activity from the prototypical activity pattern found in young adults is associated with poorer recognition performance (Düzel et al., 2011; Soch, Richter, Schütze, Kizilirmak, Assmann, Behnisch, et al., 2021) and even with performance in independent tests of memory (Richter et al., 2023). While this observation is commonly interpreted as an aging‐related reduced capability of task‐specific BOLD signal modulation, it has never before been explicitly related to the general capability to modulate functional activity, that is, also at rest. In prior resting‐state fMRI studies of neurocognitive aging, most studies focused on DMN functional connectivity, but few considered amplitude measures. Those that did found BOLD signal variability to be highly predictive of chronological age (Garrett et al., 2010; Grady & Garrett, 2014; Soch et al., 2022; Xing, 2021). Moreover, although there are both regions showing reductions and those that show increases in signal variability, regions showing reduced age‐related signal variability make up the majority (Grady & Garrett, 2014).

Here, both reduced encoding‐related deactivation within the DMN *and* generally lower modulation of the DMN BOLD signal during rest in old age were observed in the same sample of healthy young and older adults. Surprisingly, we found that older adults showed a higher amplitude of low‐frequency modulations within the hippocampus and parahippocampal gyrus compared to younger adults. Local differences in cortical amplitudes of signal fluctuations, especially in the hippocampus and parahippocampal cortex, have been reported repeatedly across age and patient groups (Bråthen et al., 2018; Han et al., 2011; Xi et al., 2012). However, despite showing that local amplitude of fluctuation can even predict cognitive performance improvements in healthy older adults (Bråthen et al., 2018), they remain inconclusive as to what exactly such measures reflect in terms of neurophysiological functioning.

### 4.2 The relationship between midline DMN regions at rest and episodic memory

When directly assessing the voxel‐wise relationship between resting‐state amplitude and successful encoding‐related activity within DMN regions (Section 3.3.3), we found a negative relationship between mPerAF and SME in the precuneus in young adults only, that is, higher resting‐state mPerAF was associated with more pronounced encoding‐related deactivations in the young participants. This indicates that the amplitude changes during rest and the task‐related modulation of the BOLD signal are related at least in some parts of the DMN. It further supports the general observation that older adults have relatively lower control of the modulation of functional activity within DMN structures. Notably, the—overall lower—mPerAF amplitudes and successful encoding‐related reduced deactivations in older adults were not correlated within the older group, suggesting that, compared to young adults, older adults also show, to some extent, a *disrupted relationship* between DMN activity fluctuations at rest and task‐related deactivations. This observation is in line with the notion that with increasing age, a lower situation‐specific modulation of DMN activity can be observed (Malagurski et al., 2020). It must be cautioned, however, that the lower inter‐individual BOLD amplitude of fluctuation may itself also have indirectly contributed to our inability to detect correlations between mPerAF and SME within the DMN in older adults, due to the lower range of values.

In a correlational analysis between mean contrast estimates in DMN ROIs and memory performance indices (Section 3.3.4), we found a positive correlation between mean resting‐state mPerAF in DMN and mPFC/ACC, and PCC ROIs and memory performance (Table 6). This suggests that older adults who generally show higher amplitudes of BOLD fluctuations at rest within the DMN also show better ability in incidental as well as intentional episodic memory encoding. In young adults, such a relationship was only observed for the PCC ROI, indicating that the relationship between resting‐state mPerAF and memory performance was weaker.

In older adults, higher resting‐state mPerAF during rest may reflect a relatively preserved ability to modulate functional activity within the DMN. It could thus be considered a potential manifestation of successful brain maintenance (Nyberg et al., 2012). Our finding is in line with a study reporting an association between reduced DMN connectivity at rest and memory deficits (Ward et al., 2015). These findings support the potential of using resting‐state measures as a marker not only for brain pathology (Chen et al., 2021; Jones et al., 2011; Mevel et al., 2011), but also for the spectrum of healthy aging. However, it should be stressed that we did not investigate connectivity but percent amplitude of fluctuation. This measure is more similar to task‐related differences in percent signal change and can be integrated into multimodal voxel‐wise analyses with task fMRI contrasts such as the SME. Unfortunately, most studies of age differences in resting‐state amplitude measures did not assess a potential relationship with cognitive performance (La et al., 2016; Xing, 2021). Thus, comparisons to existing literature have to remain tangent. A study in participants with Alzheimer's disease risk states (subjective cognitive decline and amnestic mild cognitive impairment) revealed regional differences in fractional ALFF (fALFF), where positive correlations were observed between left precuneus fALFF and MMSE scores as well as verbal fluency (Cui et al., 2021). The authors interpreted this finding as fALFF being indicative of the degree of disruption in functional networks.

Additionally, our correlations with ROI‐wise mean signal estimates revealed a negative relationship between the successful encoding‐related activity (i.e., SME) and memory performance in the precuneus ROI for older adults only (Figure 8b). The relationship was such that lower deactivations during successful memory encoding in the fMRI task were associated with poorer memory performance in another memory task (VLMT) 1 day later. In line with the above‐proposed interpretation of the positive relationship between the sign‐free mPerAF and memory performance, this could also be interpreted as reflecting that a relatively preserved ability to modulate functional activity in the precuneus (here: higher deactivation during encoding) is associated with better memory performance.

### 4.3 Hippocampal volume is not linked to DMN activity at rest, during successful encoding, nor to behavioral measures of episodic memory performance

Despite the large sample size, neither the SME, nor resting‐state mPerAF, nor behavioral memory performance showed any association with hippocampal volume, tested voxel‐wise and with mean values per DMN ROI. Therefore, our hypothesis that the relatively higher encoding‐related activity within midline DMN structures in older adults may reflect compensatory, hippocampus‐independent encoding strategies, could not be supported. Our hypothesis was based on the observation that the hippocampus plays an essential role in associative and episodic long‐term memory formation (e.g., Baars & Gage, 2010; Martin, 2018; Moscovitch et al., 2005), while—at the same time—it is less involved or even suppressed during the formation of novel associations that are highly congruent with existing knowledge (Tse et al., 2007, 2011; van Kesteren et al., 2012). Instead, midline regions of the DMN, the precuneus (Schott et al., 2019) and mPFC (van Kesteren et al., 2014), play a central role. This differentiation between classical hippocampus‐dependent encoding and more mPFC‐dependent encoding in the case of schema‐congruency goes as far as a double dissociation (van Kesteren et al., 2013).

Evidence against such an atrophy‐compensation hypothesis has been reported in several other studies (see McDonough et al., 2022, for a review). McDonough and Madan (2021) suggest that older adults with higher cognition at an old age may instead have generally higher structural complexity and more efficient brain function, thus, lower activity. Aside from the compensation hypothesis, the consistent null effect of hippocampal volume on the SME was surprising, because hippocampal volumes differed significantly between age groups with smaller volumes in the older participants who also showed poorer performance in this task. This suggests that the differences in memory performance cannot be significantly attributed to the hippocampus but are due to other functional processing differences. Notably, in an extensive review of the relationship between hippocampal volume and memory ability in healthy adults across the lifespan, Van Petten (2004) reports that, despite this idea being stuck in the expectations of researchers on neurocognitive aging ever since reports of patient H.M. (Gold & Squire, 2005), the evidence for a positive relationship between hippocampal size and episodic memory ability in older adults is relatively weak, and more recent studies suggest that it may be evident only in large cohorts and subfield‐specific (Kirchner et al., 2023; Raschick et al., 2022). Regarding functional neuroimaging, evidence supports that age‐related memory impairments are less due to differences in hippocampal involvement than to a loss of parietal deactivation (Miller et al., 2008). Moreover, in older individuals at increased risk for Alzheimer's disease, the association of hippocampal novelty responses with CSF Tau concentrations was found to be independent of hippocampal volumes (Düzel et al., 2018).

### 4.4 Implications for multi‐modal neuroimaging and clinical research

In this study, we followed a voxel‐wise multi‐modal data analysis approach to (i) account for age‐related regional differences in GMV, which affected resting‐state mPerAF more than the SME, and (ii) to enable the voxel‐wise analysis of the relationship between resting‐state amplitudes and SME. Voxel‐wise multimodal analyses are to this date relatively rare, which may be attributable to the previous lack of easy‐to‐use tools. A toolbox termed *Biological Parametric Mapping* (Casanova et al., 2007) was available for SPM5, but has not been updated for the subsequent versions of SPM. Perhaps due to a lack of compatibility with SPM8 and SPM12, not many studies have made use of BPM, but those that did could show the importance to account for regional GMV differences when comparing groups with differences in cortical morphology, for example, healthy older adults and individuals with Alzheimer's disease (Agosta et al., 2012; Jones et al., 2011).

For the current study, we created a novel multi‐modal analysis toolbox designed for use with SPM12. We hope that this toolbox will encourage others to make use of the opportunity to account for confounding factors that often occur in between‐group comparisons. The toolbox can also be applied to assess the voxel‐wise influence of within‐group individual differences on the regional BOLD signal, and its use is not limited to GMV or resting‐state fMRI as employed here, but it could also be applied, for example, to white matter lesions (using, e.g., FLAIR images), or regional differences in metabolism (as assessed, for example with PET; Schlagenhauf et al., 2013).

While our study focused on the relationship between the voxel‐wise and ROI‐wise resting‐state mPerAF and subsequent memory responses in healthy older adults, it may be of interest to further evaluate the response pattern of midline DMN structures in risk states for dementia. For example, a recent investigation in the DELCODE (Jessen et al., 2018) revealed that the precuneus novelty response exhibits an inversed U‐shaped response pattern across the Alzheimer's disease risk spectrum from healthy older adults to individuals at risk (SCD, MCI) to patients with early‐stage Alzheimer's dementia (Billette et al., 2022), and it may be promising to look at the relationship to local tau pathology (Düzel et al., 2022).

### 4.5 Limitations

One of our considerations behind the hypothesis that DMN hyperactivation during successful encoding may be negatively related to hippocampal volumes in older adults was the idea of a compensatory strategy. Although our data did not indicate a relationship between hippocampal volume and DMN hyperactivation during successful encoding, it cannot be fully excluded based on our data. To more specifically test this compensation hypothesis, future research should use explicit encoding tasks, manipulate encoding strategies (e.g., as in Brod & Shing, 2019; Ryan et al., 2020) and assess the relationship to hippocampal and regional GMV loss.

A further limitation may be the memory test employed. Here, we assessed functional activity during successful incidental visual episodic memory encoding based on later assessed explicit recognition‐confidence ratings. A different type of memory test, for example, cued recall or free recall (which would also have required a different encoding task) may have led to different results regarding effects on the SME (Uncapher & Wagner, 2009). Moreover, an encoding task more specifically aimed at testing the compensation hypothesis by directly inducing prior knowledge‐related encoding and comparing it to another type of intentional episodic encoding (e.g., like Brod & Shing, 2019) might bring different results.

Another limitation that should be mentioned is the fact that some age differences in (particularly resting‐state) fMRI may be related to neurovascular dysfunction. A variant of resting‐state fluctuation amplitudes have been linked to vascular function (Kannurpatti et al., 2012, 2014), and controlling for this measure in fMRI activity during a sensory‐motor task removed part of the age differences in task fMRI (Tsvetanov et al., 2015; but notably not in the motor cortices and occipital poles). It is therefore possible that age differences in mPerAF at rest may in part reflect neurovascular differences. However, as older adults did not show a direct relationship between resting‐state mPerAF and encoding‐related fMRI (i.e., SME), it can be argued that mPerAF cannot be reduced to a measure of neurovascular functioning alone, because in that case, the relationship between mPerAF and SME would have been expected to be significant in older adults. Moreover, as a proxy for neurovascular dysfunction, we included WMLV as a covariate in our models, because T2 hyperintensities are typically of vascular origin in otherwise healthy individuals (Gaubert et al., 2021; Saji et al., 2015; Tomimoto, 2015).

## 5 CONCLUSION

Our findings suggest that overall activity modulation is either generally reduced or shows higher inter‐individual variability in older age. The latter interpretation is additionally supported by the lack of a voxel‐wise association between resting‐state mPerAF and SME in older as compared to younger adults. Furthermore, in older adults, we found that resting‐state amplitude of fluctuation of the DMN, especially midline structures like the mPFC, was positively related to episodic memory performance. Beyond the scope of the present study, the multi‐modal neuroimaging analyses employed here show the relevance of assessing or at least controlling for the relationships between different modalities such as GMV and resting‐state BOLD amplitudes when investigating task‐related group differences with fMRI. This approach seems promising for illuminating cognitive aging with a more comprehensive assessment of the complex interplay between brain structure, network function, and cognition, and could further be enriched by modeling encoding strategies based on behavioral assessments.

## AUTHOR CONTRIBUTIONS

**Jasmin M. Kizilirmak, Björn H. Schott:** Conceptualization; **Anni Richter, Hannah Feldhoff, Larissa Fischer, Lea Knopf, Matthias Raschick, Annika Schult:** Investigation; **Jasmin M. Kizilirmak, Joram Soch, Björn H. Schott:** Methodology; **Jasmin M. Kizilirmak, Joram Soch, Anni Richter, Björn H. Schott, Renat Yakupov:** Formal analysis; **Anni Richter, Emrah Düzel, Björn H. Schott:** Project administration; **Joram Soch, Hartmut Schütze:** Software; **Anni Richter, Emrah Düzel, Björn H. Schott:** Supervision; **Jasmin M. Kizilirmak:** Visualization; **Jasmin M. Kizilirmak:** Writing – original draft; **Joram Soch, Anni Richter, Björn H. Schott, Anne Maass:** Writing – review & editing; **Emrah Düzel:** Resources; **Anni Richter, Emrah Düzel, Björn H. Schott:** Funding acquisition.

## FUNDING INFORMATION

This study was supported by the State of Saxony‐Anhalt and the European Union (Research Alliance “Autonomy in Old Age” to Anni Richter, Emrah Düzel, and Björn H. Schott) and by the Deutsche Forschungsgemeinschaft (SFB 1436, TP A05 to Björn H. Schott; DFG RI 2964‐1 to Anni Richter). The funding agencies had no role in the design or analysis of the study. The authors have no conflict of interest, financial or otherwise, to declare.

## Supporting information


**Data S1:** Supporting Information

## Data Availability

The raw MRI data are currently not publicly available, but will be shared with researchers upon reasonable request. All other data are available as follows: mPerAF, GMV, and SME contrast maps for all participants can be found in a NeuroVault repository at https://neurovault.org/collections/XDYPPLTD/. The participant information (e.g., group membership, age, sex), MATLAB and R scripts are available at https://osf.io/gfw85/, where we also published the original study protocol. The multimodal neuroimaging toolbox can be found at https://github.com/JoramSoch/MMA.

## Footnotes

^1^
Due to this procedure, we did not apply any common denoising strategies for resting‐state data such as regressing out signals from white matter and CSF for the models reported here. As a control, we also calculated our models with denoised resting‐state data (using the denoising module implemented in the CONN toolbox; Whitfield‐Gabrieli & Nieto‐Castanon, 2012). The pattern of results did not change qualitatively, and age differences actually became larger (for the original data and code see denoising script, thresholded NIFTI files and results tables in our OSF repository: https://osf.io/nzh2g/). Somewhat unexpectedly, we also found more pronounced age differences in white matter and CSF in the denoised data, *despite* regressing out white matter, CSF and six subject‐wise motion parameters. Therefore, we chose to report the non‐denoised mPerAF results here.

^2^
Since the RESTplus toolbox only provides 4 default masks, a group‐level mask fitting the dimensions and voxel sizes of our preprocessed task‐based fMRI was generated and added to the mask directory. Additionally, the parallel processing mode using outdated MATLAB commands had to be switched off.

^3^

https://github.com/JoramSoch/MMA.

^4^
In our terminology, all voxel‐wise imaging variables are regarded as different “modalities”, although resting‐state mPerAF and SME contrast maps were both acquired via functional MRI.

^5^

https://osf.io/nzh2g/.

^6^

https://neurovault.org/collections/XDYPPLTD/.

^7^
Please note that there were slight adaptations relative to the models proposed in our previously published study protocol, because of the dependencies between models A–C and 1–3 (in case a confounding factor had an influence, it was added to the models 1–3). All changes are explained in Table S1.

^8^
To test for confounds related to high collinearity, we computed the variance inflation factor (VIF) for HC_vol and TIV. The VIF was 1.7 for young adults and 1.4 for older adults. As VIFs below 2.5 are considered unproblematic (Johnston et al., 2018), we decided to keep our models as close as possible to those pre‐registered on our OSF repository.
