# Supplementary material for: The relationship between resting‐state amplitude fluctuations and memory‐related deactivations of the default mode network in young and older adults
Source: Hum Brain Mapp. 2023 Apr 13;44(9):3586–609. doi: 10.1002/hbm.26299 (PMC10203811; doi:10.1002/hbm.26299)
Supplement: Supplementary file 1 — Data S1: Supporting Information [file HBM-44-3586-s001.pdf]

RUNNING HEAD: DMN Structures and Memory Encoding in Older Adults

# – Supplementary Material –

The relationship between resting-state amplitude fluctuations and memory-related deactivations of the Default Mode Network in young and older adults

Jasmin M. Kizilirmak[#,1,2], Joram Soch[1,4], Hartmut Schütze[5,6], Emrah Düzel[5,6,7], Hannah Feldhoff[3], Larissa Fischer[3], Lea Knopf[3], Anne Maass[5], Matthias Raschick[3], Annika Schult[3], Renat Yakupov[5], Anni Richter[*,3], Björn H. Schott[*,#,1,3,7,8]

1) Cognitive Geriatric Psychiatry, German Center for Neurodegenerative Diseases, Göttingen, Germany

2) Neurodidactics and NeuroLab, Institute for Psychology, University of Hildesheim, Hildesheim, Germany

3) Leibniz Institute for Neurobiology, Magdeburg, Germany

4) Bernstein Center for Computational Neuroscience, Berlin, Germany

5) German Center for Neurodegenerative Diseases, Magdeburg, Germany

6) Institute for Cognitive Neurology and Dementia Research, Otto-von-Guericke-University, Medical Faculty, Magdeburg, Germany

7) Center for Behavioral Brain Sciences, Magdeburg, Germany

8) Department of Psychiatry and Psychotherapy, University Medical Center Göttingen, Göttingen, Germany

[*] These authors contributed equally to this work.

[#] **Correspondence** should be addressed to JMK (jasmin.kizilirmak@dzne.de) or BHS (bjoern-hendrik.schott@dzne.de).

1. Supplementary Methods

On 2021-08-29, we published our study protocol as a form of preregistration on the Open Science Framework in a repository (https://osf.io/gfw85/), simultaneously announcing it via Twitter (https://twitter.com/JKizilirmak/status/1431995887060983808). In our Methods/Data Analysis section, there were some dependencies of analyses (models 1-3 are dependent on outcomes of models A-C). We already pointed out that we might need to adapt some models based on whether certain potential confounding factors (such as sex or scanner) had an influence. However, to enhance readability, we opted for describing only the final models in the main manuscript. Here, we list the previously proposed models, the final models used, and the rationale behind adaptations for transparency. Models without changes (models A & B) are not listed.

Table S1. List of adapted models used for the neuroimaging analyses and rationale behind changes (preregistered versus final version)

Model	Case	Formula	Rationale
**C**	Prereg	GMV ~ scanner(Skyra, Verio) + sex(m,f) + scanner*sex + TIV	Age was added exploratorily, as age effects on GMV were not assessed in any of the other models.
	Final	GMV ~ scanner(Skyra, Verio) + sex(m,f) + scanner*sex **+ age** + TIV	
**1**	Prereg	mPerAF ~ age_group + HC_vol + **mPFC_GMV + PreCun_GMV** + age + TIV	*Scanner* was additionally included as a categorical variable, because it had an effect on mPerAF as found with model A. *GMV* was included as a voxel-wise imaging covariate instead of two mean ROI values to account for any voxel-wise GMV differences. White-matter lesion volume (WMLV) was included based on a reviewer's comment.
	Final	mPerAF ~ age_group **+ scanner** + HC_vol + TIV + **WMLV** + age **+ GMV**	
**2**	Prereg	SME ~ age_group + HC_vol + GMV + age + TIV	*Scanner* was included, because it had an effect on mPerAF and we wanted the models for task-fMRI SME and rs-fMRI mPerAF to be completely comparable. WMLV was included based on a reviewer's comment.
	Final	SME ~ age_group **+ scanner** + HC_vol + TIV + **WMLV** + age + GMV	
**3**	Prereg	SME ~ age_group + mPerAF + age	See rationale of inclusion of *scanner* in model 1. GMV was included based on a reviewer's comment.
	Final	SME ~ age_group **+ scanner** + age + mPerAF + **GMV**	

2. Supplementary Results

2.1. Age-group comparison of white matter lesion volume

A comparison of white matter lesion volume (WLMV; see Figure S1) for both age groups revealed significant differences between young (mean = 0.14, SD = 0.16) and older adults (mean = 1.67, SD = 2.44) according to a Wilcoxon rank-sum test [$W = 10548$, $p < .001$].

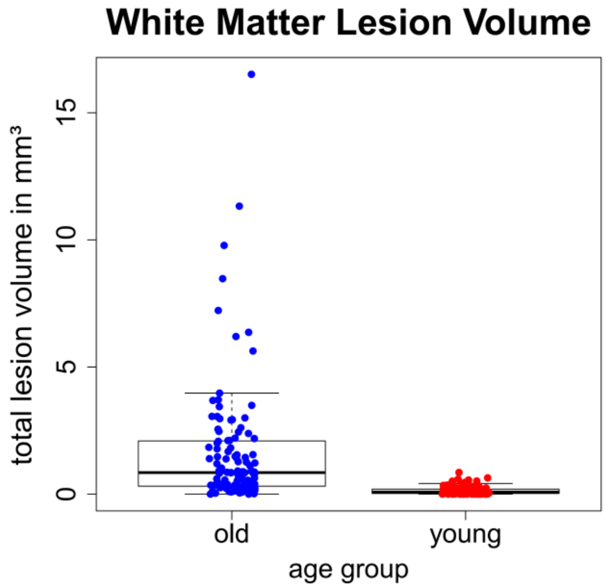

Figure S1. White matter lesion volume. Depicted are boxplots, single dots representing the participants. The box length represents the interquartile range (IQR), the ends of the whiskers are at ± 1.5 IQR respectively. The bold black bars represent the median.

2.2. Age-group comparison of hippocampal subvolumes

Here, we report the results of additional analyses we performed with subregions of the hippocampus (HC) instead of just total HC volume. These were defined as described in a doctoral dissertation which built upon a study by Maass and colleagues (Heil, 2020; Maass et al., 2014). Input regions of HC are specifically associated with memory encoding and encompass CA2/3, CA4 and dentate gyrus. Note that automatic FreeSurfer segmentation of the HC (Iglesias et al., 2015) offered only a segmentation of the granule cell and molecular layers of the dentate gyrus (GC-ML-DG), and CA2 and CA3 were collapsed. Output regions are associated with memory retrieval and encompass CA1 and the subiculum (Maass et al., 2014).

Table S2. Volumes of total HC, HC subfields, and TIV in mm³

age group	total HC	HC input	HC output	TIV
young	6890.91 ± 638.65	1524.71 ± 75.26	2300.72 ± 221.22	1489.67 ± 138.61
older	6453.07 ± 593.21	1437.43 ± 160.08	2125.97 ± 213.87	1504.37 ± 141.88

To compare volumes of HC input and output regions for both age groups, we ran ANCOVAs including TIV as a covariate. Variances were modelled as equal, because Levene's tests showed no significant differences (all $p > 0.3$).

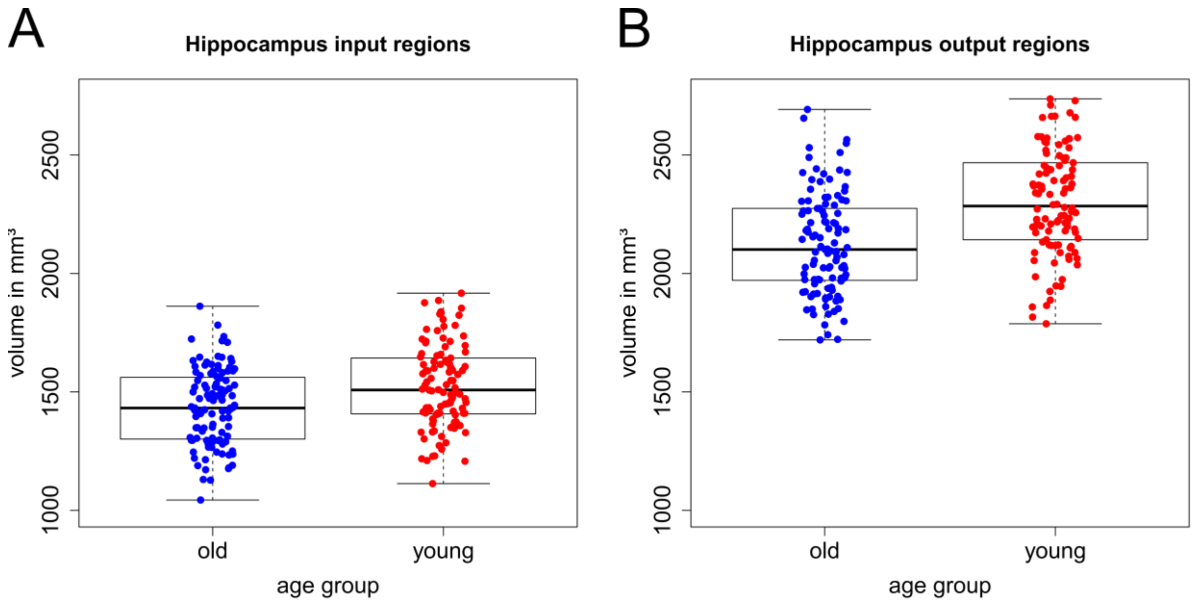

Figure S2. Volumes of the hippocampus input (CA2/3, CA4, DG; panel A) and output regions (CA1, subiculum; panel B).

As can be seen in Figure S2 (but see Table S1), age groups showed highly significant differences for both [HC input: $F(1,214) = 15.59$, $p < 0.001$; HC output: $F(1,214) = 36.97$, $p < 0.001$]. In both models, there was also highly a significant effect of the TIV covariate [HC input: F(1,214) = 14.10, p < 0.001; HC output: F(1,214) = 13.08, p = 0.001].

2.3. Confounding effects of scanner and TIV

Effect of scanner on resting-state mPerAF. The effect of scanner was such that participants scanned with Skyra showed relatively higher mPerAF in frontal and temporo-parietal white matter than those scanned with Verio (Figure S2A and Supplementary Table S2). For participants scanned with Verio, mPerAF differences in comparison to Skyra scans were mainly restricted to the cerebellum (Figure S2B). Previous studies have shown that even when scanning protocols are harmonized, different head coils may lead to biases, i.e. higher regional sensitivity for BOLD signal changes, especially in resting-state fMRI (Kaza et al., 2011; Panman et al., 2019).

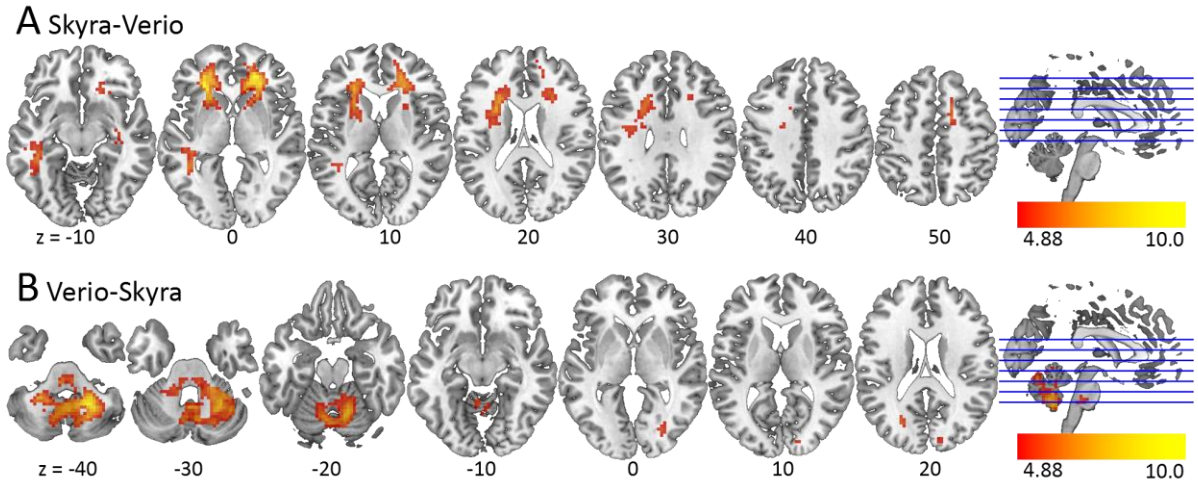

Figure S3. Effects of scanner on resting-state mPerAF. Panels A and B represent the differences between the scanners.

Table S3. Effects of scanner on mPerAF.

anatomical label	cluster size	peak p(FWE-corr)	peak T	x,y,z {mm}
**positive effect of scanner (Skyra > Verio)**				
	742	0.000	10.12	-27 32 -1
		0.000	9.44	-24 32 8
		0.000	9.00	-27 41 -1
	534	0.000	10.07	21 38 5
		0.000	9.31	18 38 -4
		0.000	9.20	27 35 -1
	207	0.000	7.84	-39 -43 2
		0.000	7.07	-42 -34 -10
		0.000	6.52	-39 -31 -1
R supplementary motor area	61	0.000	7.80	15 -1 56
R superior frontal gyrus		0.000	6.52	15 11 50
R supplementary motor area		0.001	5.75	12 -7 62
	15	0.000	6.47	33 -19 -7
		0.017	5.11	39 -19 -13
**negative effect of scanner (Verio > Skyra)**				
	1806	0.000	9.86	21 -52 -40
R cerebellum, exterior		0.000	9.47	21 -61 -25
R cerebellum, exterior		0.000	9.27	30 -52 -28
L superior occipital gyrus	32	0.000	6.88	-21 -67 26
R calcarine gyrus	25	0.000	6.53	15 -88 14
R cuneus		0.010	5.27	15 -82 29
	24	0.000	5.98	27 -76 -1
		0.012	5.19	27 -85 8

Note. Table shows 3 local maxima more than 8.0 mm apart. Anatomical labels for coordinates according to AAL3 atlas. Height threshold: T = 4.88, p = 0.000 (0.050); extent threshold: k = 10 voxels, p = 0.001 (0.000); expected voxels per cluster k = 0.703; expected number of clusters c = 0.00. Degrees of freedom = [1.0, 212.0], FWHM = 8.7 x 8.9 x 8.3 mm^3; 2.9 x 3.0 x 2.8 voxels; volume: 47654 voxels = 1751.0 resels; voxel size = 3 mm isotropic; resel = 23.72 voxels.

Effects of age and TIV on GMV. The positive effect of age (higher GMV with higher age) was bilaterally located in white matter regions (see Figure S3A and Supplmentary Table S3). It could possibly be explained by a reduction of white matter which leads to more grey matter being located in voxels containing white matter in young participants. The negative effect of age could be seen throughout the whole cortex, as to be expected (the younger, the higher GMV; see Figure S3B). The effect of TIV was positive (higher TIV, higher GMV; see Figure S3C and Supplementary Table S4). However, as described by a comprehensive study on the role of the inclusion of TIV and sex in analyses of GMV (Sanchis-Segura et al., 2019), TIV explains a high amount of variance that would otherwise be attributed to sex.

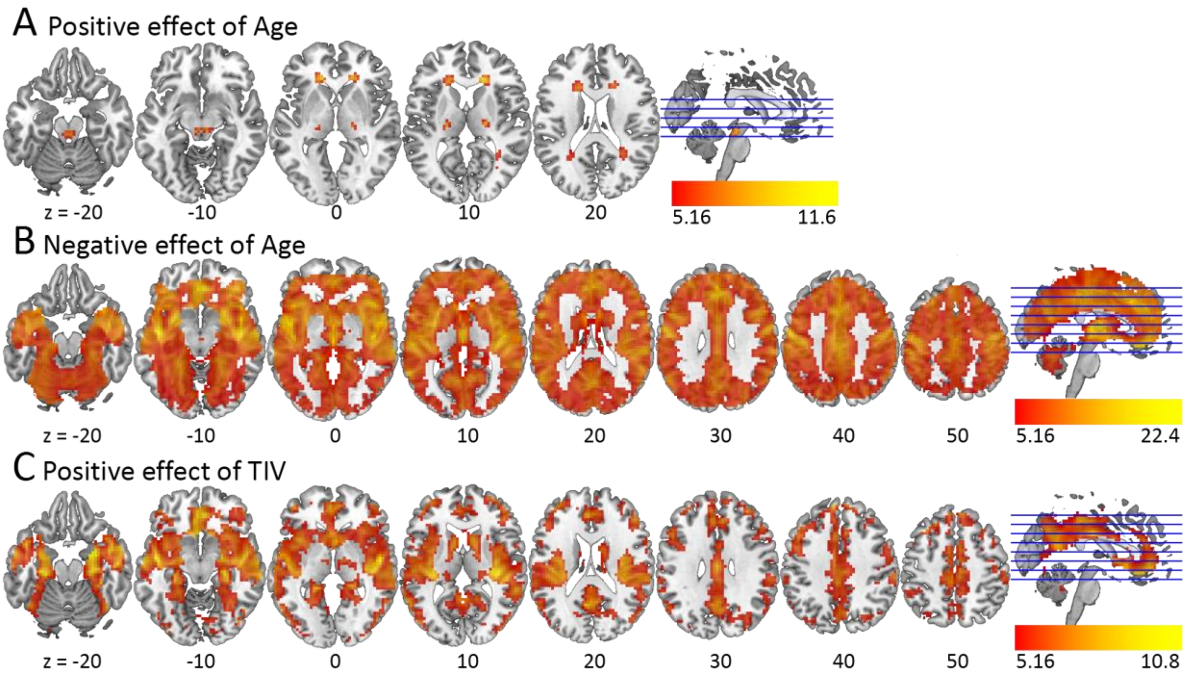

Figure S4. Effects of age and TIV on GMV. Note that the effects are masked with an inclusive mask of active voxels for SME and mPerAF combined.

Table S4. Effects of age on GMV.

anatomical label	cluster size	peak p(FWE-corr)	peak T	x,y,z {mm}
**positive effect of age**				
R thalamus, ventral posterolateral	145	0.000	11.60	18 -16 5
L thalamus, ventral posterolateral		0.000	10.59	-21 -19 8
		0.000	9.69	3 -25 -13
	109	0.000	11.48	18 32 5
	139	0.000	9.78	-15 32 -1
		0.000	8.58	-18 26 17
	55	0.000	7.62	30 -49 17
		0.023	5.32	33 -64 11
	16	0.000	6.83	15 -61 -34
		0.002	5.85	6 -49 -28
	23	0.000	6.21	-27 -52 17
	15	0.001	5.98	-15 -1 32
	18	0.002	5.77	21 -7 32

**negative effect of age**				
L superior temporal gyrus	37291	0.000	24.44	-45 -7 -7
R superior temporal gyrus		0.000	22.56	45 -10 -10
R superior frontal gyrus, medial orbital		0.000	22.37	3 35 -13

Note. Table shows 3 local maxima more than 8.0 mm apart. Anatomical labels for coordinates according to AAL3 atlas. Height threshold: T = 5.16, p = 0.000 (0.050); extent threshold: k = 10 voxels, p = 0.001 (0.000); expected voxels per cluster k = 0.680; expected number of clusters c = 0.00. Degrees of freedom = [1.0, 211.0], FWHM = 8.9 x 9.2 x 8.9 mm³; 3.0 x 3.1 x 3.0 voxels; volume: 173106 voxels = 6045.4 resels; voxel size = 3 mm isotropic; resel = 27.11 voxels.

Table S5. Effects of TIV on GMV.

anatomical label	cluster size	peak p(FWE-corr)	peak T	x,y,z {mm}
**positive effect of TIV**				
R superior temporal gyrus	13690	0.000	10.80	51 -10 -1
R superior frontal gyrus, medial orbital		0.000	10.76	3 35 -13
R amygdala		0.000	10.71	27 -1 -19
	108	0.000	7.75	33 -49 -43
		0.000	6.96	33 -61 -46
		0.000	6.59	18 -70 -40
	29	0.000	6.87	6 -46 -31
		0.000	6.40	-3 -52 -28
		0.002	5.77	15 -49 -28
L cerebellum, exterior	18	0.000	6.28	-24 -58 -31
R calcarine	17	0.000	6.18	21 -79 5
R calcarine		0.004	5.67	21 -79 14
	10	0.001	6.05	24 -58 -31
L inferior parietal gyrus	32	0.001	5.89	-42 -43 50

Note. Table shows 3 local maxima more than 8.0 mm apart. Anatomical labels for coordinates according to AAL3 atlas. Height threshold: T = 5.16, p = 0.000 (0.050); extent threshold: k = 10 voxels, p = 0.001 (0.000); expected voxels per cluster k = 0.680; expected number of clusters c = 0.00. Degrees of freedom = [1.0, 211.0], FWHM = 8.9 x 9.2 x 8.9 mm³; 3.0 x 3.1 x 3.0 voxels; volume: 173106 voxels = 6045.4 resels; voxel size = 3 mm³; resel = 27.11 voxels.

2.4. Relationship of hippocampal input and output volume to DMN activity at rest (mPerAF)

As we expected no relationship between hippocampal volume and resting-state activity, we also tested the predictive ability of the volume of HC input regions on resting-state mPerAF. To this end, we ran the same BPM analysis as in the manuscript proper under 3.4.1, only with volume of HC input and output regions instead of total HC volume. We ran the following model:

mPerAF ~ age_group(young, old) + scanner(Skyra, Verio) + HCinput + HCoutput + TIV + age + GMV,

whereby GMV was included as an imaging covariate and HCinput, HCoutput, and TIV as non-imaging covariates. The following regressors were included: Skyra_young, Verio_young,

Skyra_old, Verio_old, HCinput_young, HCinput_old, HCoutput_young, HCoutput_old, TIV_young, TIV_old, age, GMV.

With voxel-wise FWE correction, p < .05, cluster extent threshold = 10, just one small cluster of 11 voxels was found for the positive effect of HC output volume for older participants within the cerebellum. This effect does not seem meaningful and will not be interpreted.

2.5. Effects of voxel-wise GMV on resting-state mPerAF and SME

As mentioned in the main paper, GMV had significant (FEW-corrected p < .05, cluster threshold = 10) effects on mPerAF and SME (Table S7). The effect on mPerAF was seen distributed across white matter regions (Figure S4A), while the effect on SME was restricted to one very central cluster that actually suggests ventricular artifacts (Figure S4B).

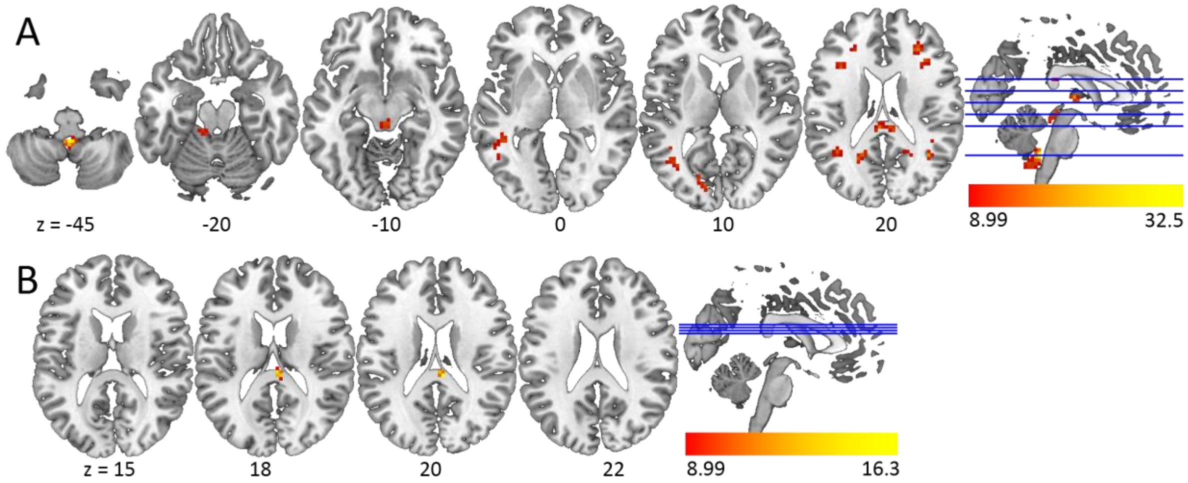

Figure S5. Effects of GMV on mPerAF (A) and SME (B). Effects are masked with an inclusive mask of active voxels for SME and mPerAF combined.

Table S6. Effects of GMV on mPerAF and SME

anatomical label	cluster size	peak p(FWE-corr)	peak F	x,y,z {mm}
**GMV on mPerAF**				
	62	0.000	32.67	0 -43 -46
		0.000	14.86	0 -52 -52
L lobule IX of cerebellar hemisphere		0.000	12.67	-9 -43 -55
R middle frontal gyrus	74	0.000	24.13	33 32 23
R middle frontal gyrus		0.000	22.44	33 26 32
		0.000	12.90	24 29 29
L precuneus	49	0.000	23.84	-12 -61 35
		0.000	17.77	-21 -58 23
		0.000	14.90	-15 -52 17
	27	0.000	23.26	15 -31 35
L angular gyrus	136	0.000	23.17	-30 -55 35
L angular gyrus		0.000	22.02	-39 -52 23
		0.000	18.98	-36 -46 35
	140	0.000	21.85	39 -55 20

R angular gyrus		0.000	19.79	36 -52 38
		0.000	19.21	30 -40 38
R thalamus, mediodorsal	11	0.000	21.58	0 -13 5
L precuneus	54	0.000	20.29	-24 -52 5
L calcarine fissure and surrounding cortex		0.000	18.80	-15 -52 8
L calcarine fissure and surrounding cortex		0.000	14.98	-12 -82 11
	13	0.000	19.44	-3 -31 20
		0.000	15.81	9 -34 20
R precuneus	33	0.000	19.14	15 -58 38
R precuneus		0.000	12.49	21 -52 17
	84	0.000	18.65	-30 26 26
		0.000	16.70	-24 23 32
		0.000	16.04	-36 20 20
R calcarine fissure and surrounding cortex	14	0.000	18.07	21 -49 5
	13	0.000	16.82	-24 -7 44
	25	0.000	16.12	-45 -43 -4
L middle temporal gyrus		0.000	13.28	-48 -37 5
L middle temporal gyrus		0.001	11.25	-51 -49 2
	12	0.000	15.58	-24 5 41
L superior frontal gyrus, dorsolateral		0.001	11.57	-18 2 50
	32	0.000	15.53	36 26 20
R inferior frontal gyrus, opercular part		0.000	13.47	33 11 35
R precentral gyrus		0.001	11.22	36 2 32
	15	0.000	15.42	0 -28 -10
	16	0.000	15.26	-15 -34 38
L middle cingulate & paracingulate gyri		0.000	13.15	-12 -40 32
L middle temporal gyrus	24	0.000	14.50	-39 -61 11
L middle temporal gyrus		0.001	11.84	-48 -58 2
L lobule IV, V of cerebellar hemisphere	16	0.000	13.65	-15 -31 -22
R middle frontal gyrus	27	0.000	12.96	33 8 47
	62	0.000	12.69	30 -4 44
		0.000	12.28	24 2 47
**GMV on SME**				
	10	0.000	16.33	6 -31 20
L precuneus	10	0.000	12.70	-15 -61 32

Note. Table shows 3 local maxima more than 8.0 mm apart. Anatomical labels for coordinates within grey matter according to AAL3 atlas. Height threshold: F = 8.99, p = 0.000 (0.050); extent threshold: k = 10 voxels.

2.6. Relationship of hippocampal input and output volume to DMN activity during memory encoding (SME)

To assess the potential predictive ability of the volume of HC input regions on encoding-related activity, we ran the same multi-modal analysis as in the main manuscript under 3.3.2, only with volume of HC input and output regions instead of total HC volume. We ran the following model:

SME ~ age_group(young, old) + scanner(Skyra, Verio) + HCinput + HCoutput + TIV + age + GMV,

whereby GMV was included as an imaging covariate and HCinput, HCoutput, and TIV as non-imaging covariates. All covariates were group-wise mean-centered. HC input was expected to have an effect, as the encompassed subregions are associated with memory encoding and thus our SME contrast, while HC output was expected to have no effect, as those subregions are associated with memory retrieval, which we did not assess with the SME (Maass et al., 2014).

The following regressors were included: Skyra_young, Verio_young, Skyra_old, Verio_old, HCinput_young, HCinput_old, HCoutput_young, HCoutput_old, TIV_young, TIV_old, age, GMV_young_Skyra, GMV_young_Verio, GMV_old_Skyra, GMV_old_Verio.

There were no effects of hippocampus volumes that survived voxel-wise FWE correction with a significance threshold of $p < .05$, cluster extent threshold = 10 voxels.

2.7. Parametric subsequent memory effects for each age group

In the main manuscript, we reported age-group differences of the SME. Here, we additionally depict the positive and negative effects of the parametric modulator (used to model the SME) for each age group separately (Figure S6 and S7).

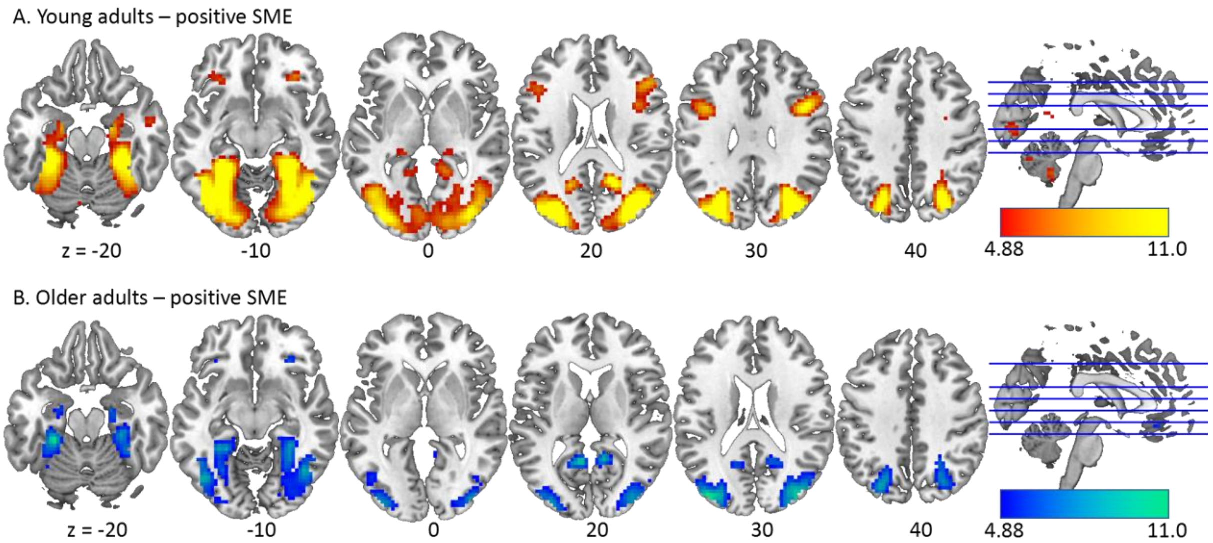

Figure S6. Positive effects of successful encoding. Data shown split for age group (A young adults, B older adults) from model 2 of the main manuscript.

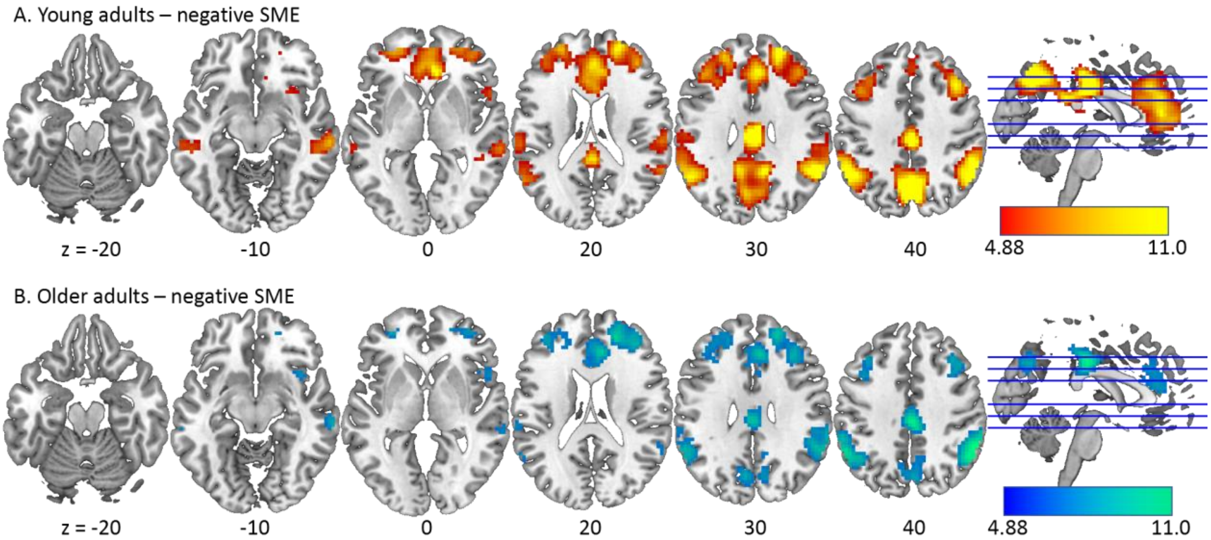

Figure S7. Negative effects of successful encoding. Data shown split for age group (A young adults, B older adults) from model 2 of the main manuscript.

2.8. Correlations between mean ROI values and memory performance

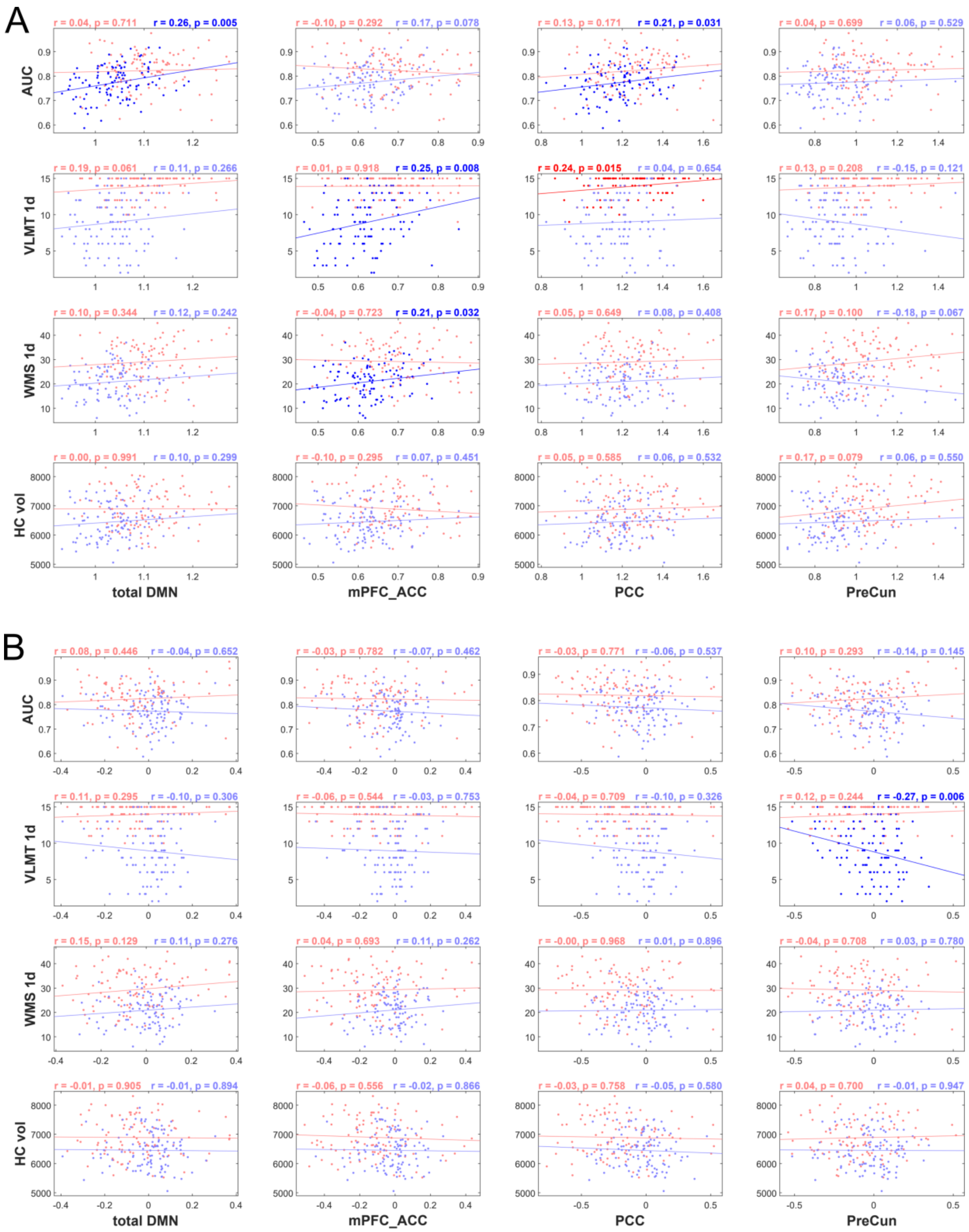

Figure S8. Relationship between DMN ROIs and memory. Correlations between DMN activity in different ROIs (panel A: resting-state mPerAF, panel B: SME contrast estimates), memory performance (AUC, VLMT, WMS), and hippocampus volume (mm^3). Young = red, older = blue. Reported are Pearson's r and p-values.

3. Supplementary References

Heil, F. J. (2020). *Der Einfluss von Einzelnukleotid-Polymorphismen verschiedener Gedächtnis- und Krankheits-assoziierter Gene auf das Volumen des menschlichen Hippocampus und seine Input- und Output-Regionen*. Otto von Guericke University, Magdeburg.

Iglesias, J. E., Augustinack, J. C., Nguyen, K., Player, C. M., Player, A., Wright, M., Roy, N., Frosch, M. P., McKee, A. C., Wald, L. L., Fischl, B., & Van Leemput, K. (2015). A computational atlas of the hippocampal formation using ex vivo, ultra-high resolution MRI: Application to adaptive segmentation of in vivo MRI. *NeuroImage*, *115*, 117–137. https://doi.org/10.1016/j.neuroimage.2015.04.042

Maass, A., Schütze, H., Speck, O., Yonelinas, A., Tempelmann, C., Heinze, H.-J., Berron, D., Cardenas-Blanco, A., Brodersen, K. H., Enno Stephan, K., & Düzel, E. (2014). Laminar activity in the hippocampus and entorhinal cortex related to novelty and episodic encoding. *Nature Communications*, *5*(1), 5547. https://doi.org/10.1038/ncomms6547
